# Vegetation and climate change at the southern margin of the Neo-Tethys during the Cenomanian (Late Cretaceous): Evidence from Egypt

**DOI:** 10.1371/journal.pone.0281008

**Published:** 2023-01-30

**Authors:** Haytham El Atfy, Clément Coiffard, Salah Y. El Beialy, Dieter Uhl

**Affiliations:** 1 Department of Geosciences, University of Tübingen, Tübingen, Germany; 2 Faculty of Science, Geology Department, Mansoura University, Mansoura, Egypt; 3 Institute of Biology, Structural and Functional Plant Diversity Group, Freie Universität Berlin, Berlin, Germany; 4 Senckenberg Forschungsinstitut und Naturmuseum Frankfurt, Frankfurt am Main, Germany; Baylor University, UNITED STATES

## Abstract

Changes in terrestrial vegetation during the mid-Cretaceous and their link to climate and environmental change are poorly understood. In this study, we use plant macrofossils and analysis of fossil pollen and spores from the Western Desert, Egypt, to assess temporal changes in plant communities during the Cenomanian. The investigated strata have relatively diverse sporomorph assemblages, which reflect the nature of parent vegetation. Specifically, the palynofloras represent ferns, conifers, monosulcate pollen producers, Gnetales, and a diverse group of angiosperms. Comparisons of both, dispersed palynoflora and plant macrofossils reveal different characteristics of the palaeoflora owing to a plethora of taphonomical and ecological biases including the depositional environment, production levels, and discrepancies between different plant organs. A combination of detailed records of sporomorphs, leaves, and charcoal from the studied successions provide new understandings of the palaeoclimate and palaeogeography during the Cenomanian and Albian-Cenomanian transition in Egypt. The mixed composition of the palynofloral assemblages reflects the presence of different depositional situations with a weak marine influence, as evidenced by a minor dinoflagellate cysts component. The local vegetation comprised various categories including herbaceous groups including ferns and eudicots, fluvial, open environments, and xeric arboreal communities dominated by Cheirolepidiaceae and perhaps including drought- and/or salt-tolerating ferns (Anemiaceae) and other gymnosperms (Araucariaceae, Ginkgoales, Cycadales, and Gnetales) as well as angiosperms. The presence of riparian and freshwater wetland communities favouring aquatic and/or hygrophilous ferns (of Salviniaceae and Marsileaceae), is noted. The wide variation of depositional settings derived from the palynological data may be attributed to a prevalent occurrence of producers in local vegetation during the early Cenomanian of Egypt. For the purpose of this work on the studied Bahariya Formation and its equivalent rock units, where iconic dinosaurs and other fossil fauna roamed, we attempt to improve the understanding of Egypt’s Cenomanian climate, which is reconstructed as generally warm and humid punctuated by phases of considerably drier conditions of varying duration.

## I. Introduction

The Cretaceous is a remarkable episode in Earth’s history that represents a period of key changes in worldwide terrestrial floras, the most significant feature is the notable phylogenetic diversification and ecological radiation of angiosperms that emerged in the Early Cretaceous [1]. The Cretaceous was a time of high (but descending) atmospheric CO_2_ (e.g., [2]) and a global greenhouse climate (e.g., [3]). According to [4], an important particularity of the Cretaceous is, tropical and polar temperatures were warmer compared to the present-day climate. As a consequence of the continuous rise in our atmospheric CO_2_ concentration since the Industrial Revolution, studying the Cretaceous climate is of particular interest as a unique analogue to potential future climate changes. Additionally, vegetal assemblages as well as other palaeoclimate proxies indicate aridification, especially for Mid-Cretaceous (i.e., 125–90 Ma) terrestrial environments [5]. Moreover, during the Cretaceous, wildfire occurred frequently worldwide, as demonstrated by numerous charcoal shreds of evidence (see [6,7]), including the Cenomanian of the studied Gebel El Dist section, Bahariya Oasis [8]. These findings could point to widespread dry and hot (at least seasonally) conditions, at least in some regions, that contributed to setting the stage for a fiery hothouse world [5].

In this context, Egypt is important as an incompletely understood region with respect to the Cretaceous climate and palaeovegaetion. The Cenomanian deposits of Egypt represent a unique example of widespread marine and non-marine conditions and consequently offer important insights into interpreting terrestrial ecosystems from this time period. These sequences include abundant and often well-preserved micro- and mega-plant fossil remains. However, palaeofloristic reconstructions from these deposits based on mega-plant fossil assemblages are still lacking, although relatively little fundamental research and a few abstracts have so far been published on the Cenomanian floras of Egypt (e.g., [9–16]).

On the other hand, several investigations on dispersed sporomorphs have been carried out (e. g., [17–21]). However, these studies mostly focused on biostratigraphy, palynofacies, source rock evaluation, and in part depositional environments, whereas detailed analyses of vegetation and climate are so far mostly lacking. Although the Cretaceous surface strata cover vast areas of Egypt, intensive weathering and oxidation destruction of organic matter, related to arid climate (e.g., [22]), make it difficult to run palynological studies on surface exposures and most of the existing data have been retrieved from subsurface material. As a result, previous contributions are mostly based on subsurface core and cuttings samples collected from exploratory wells, except for [17,23,24] who dealt with samples from surface exposures.

Previous palynological contributions hypothesized a warm tropical to subtropical, arid to semi-arid climate for the Bahariya Formation (e.g., [18,19]), and its equivalents (e.g., Galala Formation: [24], Raha Formation: [25], Maghrabi Formation: [26], Umm Sidida Formation: [27]). This hypothesis needs to be re-evaluated, as it is generally not in agreement with the occurrence of high biomass ecosystems in which abundant and diverse vertebrates roamed. This contradiction could be attributed to the lack of detailed quantitative data in many of these contributions, except for a few of them (e.g., [28]). In addition, many of the above contributions interpreted the vegetation only in a general context as belonging to the Albian-Cenomanian Phytogeographic Province (sensu [29]), and its equivalents from the coastal basins of West Africa and South America, without employing any climatic proxies or data.

**Fig 1:**
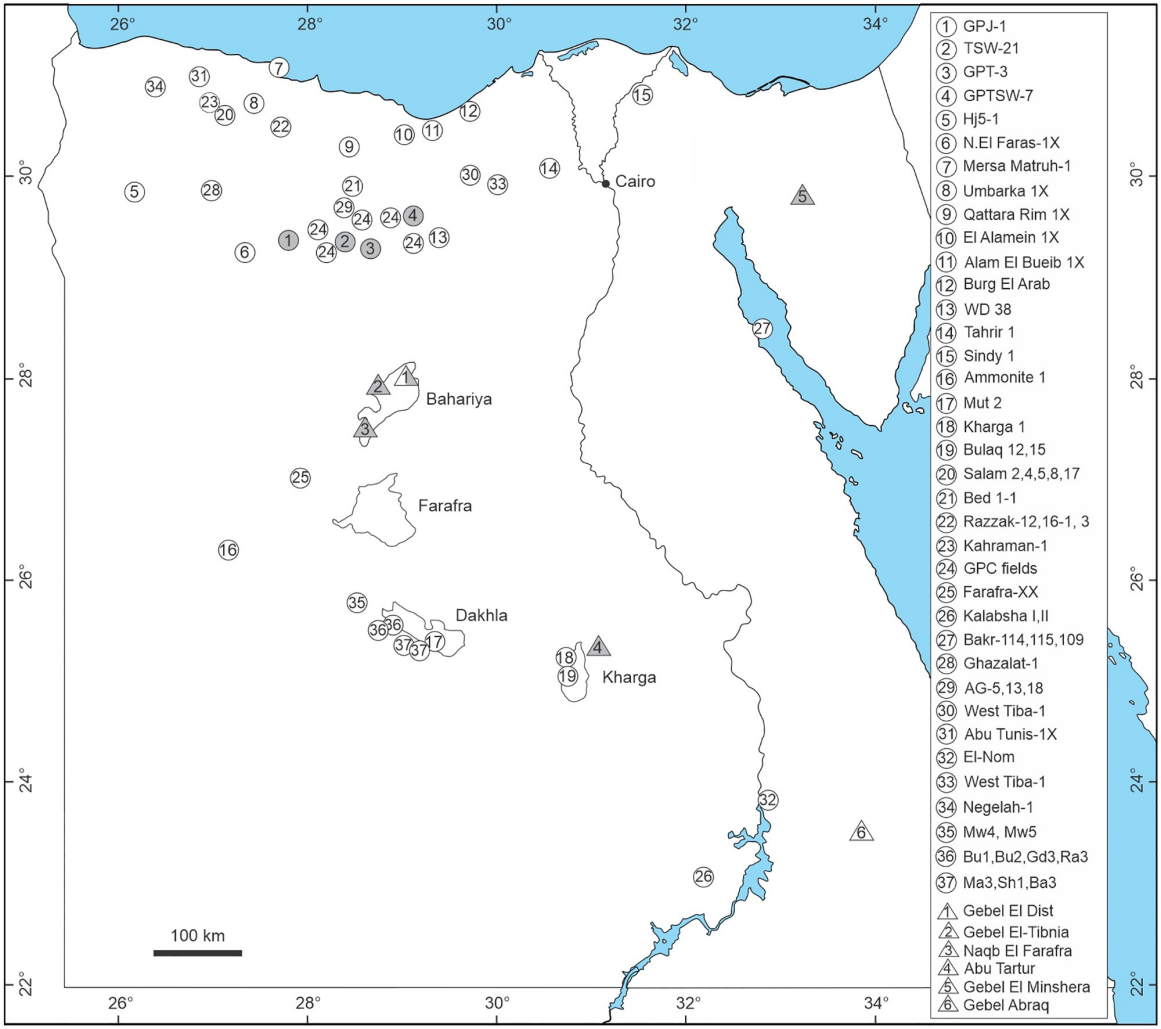
Location map of Egypt showing the studied locations and boreholes (in grey) in addition to previous Cenomanian records; numbers in circles represent subsurface occurrences of the Cenomanian, however, numbers in triangles refer to surface exposures.

From an ecosystem perspective, plants work as the bond between animals and the environment, and fossil plants offer excellent proxies to reconstruct continental palaeoenvironments and climates (e.g., [30]). Although there is an emerging consensus about global climate patterns during the Cretaceous, details about the climate development in North Africa, including Egypt at this time are poorly resolved, and estimates for continental climate are rather incomplete. A careful review of the previous contributions on palynology and megaflora during this time window in Egypt was essential as many issues concerning the climate and vegetation remain equivocal. It became more important to reconcile the two disciplines and to better understand the vegetation dynamics and climate changes during the Cenomanian of North Africa with a special focus on Egyptian deposits (Fig 1).

This current work deals with the palynofloras (retrieved from 4 wells) and macroflora (a selection curated in the collections of the Museum für Naturkunde in Berlin, Germany) from the Cenomanian deposits of the Western Desert, Egypt (Table 1), as well as Gebel El Minshera section in Sinai, which has been also studied (Fig 1). Published charcoal data are also employed to reconstruct the source vegetation and floristic composition and contribute to the evolution of the vegetation at the southern shore of the Tethys during the Cenomanian in N-Africa (Egypt). Further research is still required to improve the current knowledge about vegetation and climate changes on local and regional scales. Finally, we try to reconstruct the vegetation in which dinosaurs and other animals dwelled, to improve our understanding of the biomes which produced enough plant biomass to sustain large sauropods and different large predators feeding on these herbivores.

**Table 1:** List of the studied material and their geographic data.

Location	Geographic data	Number of samples
**A. Subsurface boreholes**:		
GPJ-1 well	Latitude 29° 32’ 08.77” & Longitude 28° 08’ 58.40	5
TSW-21 well	Latitude 29° 34’ 09.49” & Longitude 28° 30’ 10.41”	13
GPT-3 well ([73])	Latitude 29° 36’ 10.681” & Longitude 28° 36’ 43.733”	4
GPTSW-7 well ([72,62])	Latitude 29° 33’ 39.81” & Longitude 28° 31’ 36.35”	34
**B. Surface exposures and collections**:		
Gabal El Dist ([8])	north of the Bahariya Oasis, Western Desert	50
Gebel El Minshera ([33])	40 km to the Southeast of Koeniguer’s locality at Gebel Maghara, Sinai	1
Naqb El Farafra ([40]) Specimens: B0001, B0005, B0007, B0008, B0009, B0010, B0011, B0012, B0020, B0067, B0068, B0069, B0070, B0072, B0109, B0110, B0115, B0116, B0117, B0155, B1553, MB.Pb.2022/0909, MB.Pb.2022/0910, MB.Pb.2022/0915, MB.Pb.2022/0930, MB.Pb.2022/0942, MB.Pb.2022/0970, MB.Pb.2022/0971, MB.Pb.2022/0972	south of the Bahariya Oasis, Western Desert	29
Gebel El-Tibnia ([40]) Specimens: MB.Pb.2022/1019, MB.Pb.2022/1034, MB.Pb.2022/1034, MB.Pb.2022/1044, B0006, B0022, B0023, B0015, MB.Pb.2022/1014	north of the Bahariya Oasis, Western Desert	9
Abu Tartur ([71]) Specimens: B1078, B1432, B1433, B1434, B1435, B1531, B1533, MB.Pb.2022/1046, MB.Pb.2022/1047, MB.Pb.2022/1048, MB.Pb.2022/1049, MB.Pb.2022/1050	north of the Kharga Oasis, Western Desert	12

### 1.1. Background

Historically, plant remains from the Cenomanian of Egypt were repeatedly recorded since the last millennium, however [15], reported the richest megafloral assemblage. They studied plant remains of the Cenomanian from the Bahariya Formation, at the so-called mangrove-dinosaur site [31,32] located about 14 km northwest of Gebel El Dist. Their assemblage indicated a warm climate and is considered an angiosperm-dominated flora (55 leaves, 3 stems, 3 fruits, and a single inflorescence) with four pteridophytes belonging to three families, 9 gymnosperms of 5 families, and 49 angiosperms of 18 families. The latter group is dominated by 46 dicots (16 families) and only three monocot morphotypes. Moreover [14], identified a similar assemblage recorded in the Cenomanian Bahariya Formation, as originating from lagoonal and intertidal deposits. However, the occurrence of freshwater taxa *Nelumbites* and floating aquatic ferns in saltwater-induced environments, indicate that freshwater ponds pertain to the areas of paralic sedimentation. They (op. cited) assumed that the area of study was rather dry and warm during the Cenomanian. Later on [16], studied fern axes (stems or rhizomes) from Gebel Ghorabi and Gebel El Dist (Bahariya Oasis) and interpreted them to signify a swampy palaeoenvironment with a mangrove based on sedimentological and palaeobotanical data.

Palynological research focussing on the Cenomanian deposits in Egypt was initiated by [17] on the Gebel Dist at the Bahariya Oasis and continued until the recent work of [21]. However, the first study tackling Cenomanian macroflora in Egypt in detail is that of [11] with numerous subsequent studies until the most recent one of [33] on Gebel El Minshera in Sinai. Unfortunately, and in order to maximize the unique potential of this resource and avoid the pitfalls of the previous interpretations of the palaeoclimate, it is clearly necessary to develop a scheme which had the advantage of having detailed information on the palaeoclimate, vegetation, and ecosystem development, which is so far mostly lacking. Plant macro-remains studied by [16] and [34] from the Bahariya Formation probably represent a mangrove palaeoflora, an interpretation supported by the existence of typical mangrove-dwelling crabs in the same strata [32].

The Bahariya Formation is also prominent for its magnificent yield of vertebrate fossil remains, including important dinosaurs, such as the carnivorous *Spinosaurus* and *Carcharodontosaurus*, along with the herbivorous *Aegyptosaurus* and *Paralititan* (e.g., [31,35–38]). Moreover, a wide variety of continental vertebrates (aquatic lungfishes, turtles, crocodiles, and dinosaurs), and marine vertebrates including sharks, plesiosaurs, and the sea serpent *Symoliophis*, along with lamellibranchiates, ammonites, and crustaceans (e.g., [31,32,39–41]) are also known from the Cenomanian deposits in the Egyptian Western Desert.

## 2. Geological and stratigraphic setting

### 2.1. Cenomanian in Egypt: Geology and facies distribution

**Fig 2:**
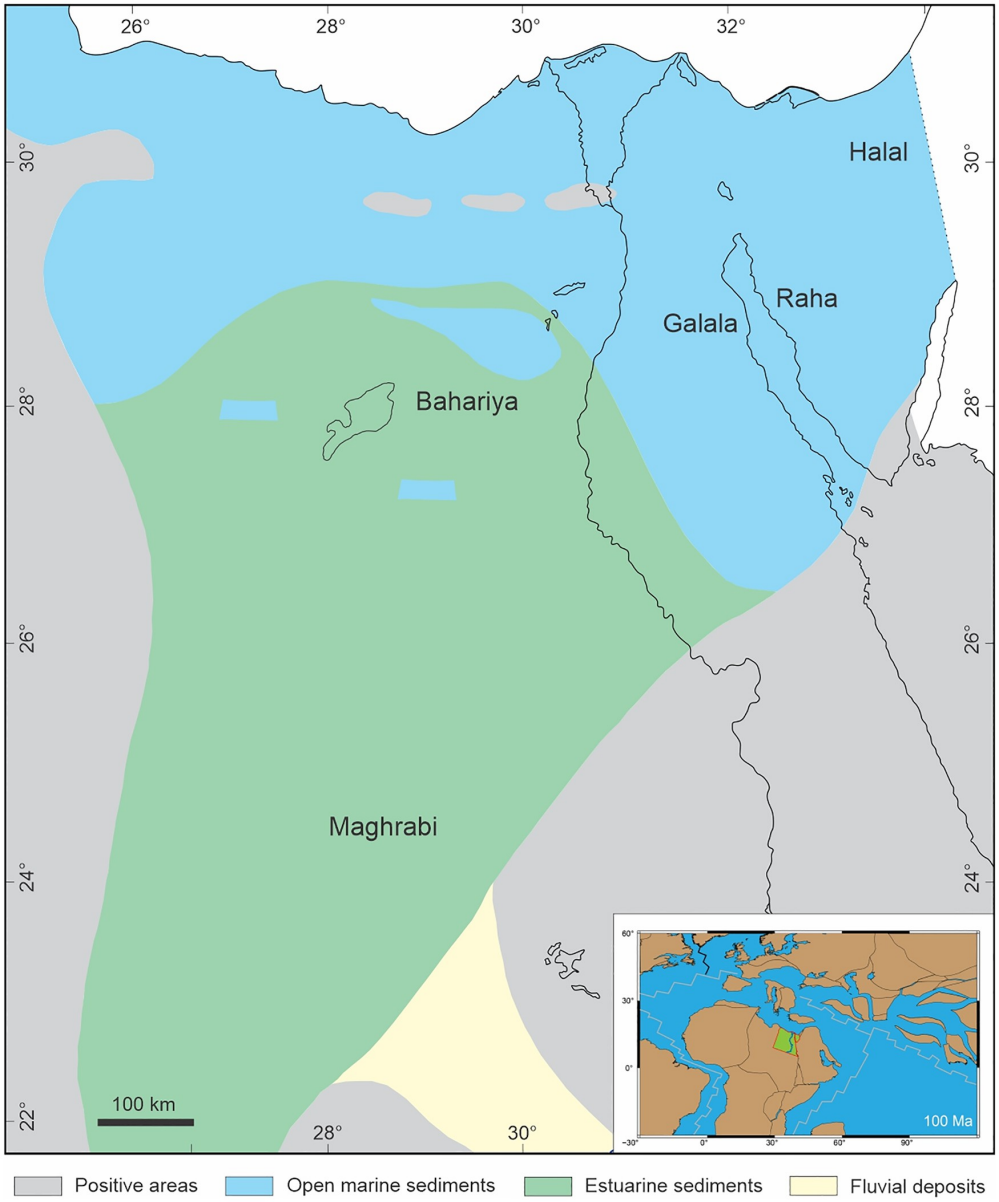
Egypt during the Cenomanian time, simplified and re-drawn after [43]. The inset box shows the palaeogeographic position of Egypt 100 Ma ago, constructed using the palaeogeographic data of [44] and drawn using Generic Mapping Tools [45].

The Cenomanian flooding in the southern margin of the Neo-Tethys Ocean characterizes the first major marine transgression over the hinterland leading to the deposition of shallow-water sediments over fluvio-marine siliciclastic Early Cretaceous deposits [42]. During the Cenomanian, a marine transgression covered most of Sinai, the Gulf of Suez, and northwest Egypt (Fig 2). In late Cenomanian, the transgression pushed southward to form a narrow passageway that lay between the Arabo-Nubian massif and the elevated Kufra basin, this passageway seems to have formed a veritable estuary in which marginal marine conditions prevailed [43].

In northern Sinai, these deposits comprise nearly solely carbonates displaying pelagic facies of rather deep water. Southwards, clastics become increasingly abundant over carbonates and the total thickness falls off gradually. According to [46], two facies were recognized for the Cenomanian transgression. The deeper marine facies of the Halal Formation in northern Sinai characterizes an incessant transgressive phase without foremost regressions. The shallow marine facies of the Raha and Galala formations in the Gulf of Suez region imitates a substantial detrital influx [47]. Wherever exposed, Cenomanian strata lie above the Malha or Risan Aneiza formations with no break in sedimentation. Intermittent sea-level fluctuations are specified in the western side of the Gulf of Suez by rapid facies changes of carbonates interchanging with sandstones [47]. In an estuary, which is pushed to the south, the fluviatile-deltaic-marine sediments of the Bahariya Formation were deposited. Further south, in the Dakhla Basin, this unit grades into the Maghrabi Formation which reflects less marine influence [43,46].

### 2.2. Lithostratigraphic framework of the Cenomanian in Egypt

#### 2.2.1. Halal Formation

The Halal Formation, at its type locality in Gebel Halal (north Sinai), is 450 m thick and made up of carbonate rocks with minor shales [48]. Its fossil content comprises mainly echinoids, rudists, oysters, and ammonites, such as *Neolobites vibrayeanus*, which represents a diagnostic index taxon for the upper Cenomanian in the Middle East and Egypt (e.g., [49]).

#### 2.2.2. Raha Formation

The Raha Formation was introduced by [50] for a 70–120 m thick succession in Gebel Raha, western Sinai. It is a heterogeneous unit composed of yellow sandstones, dolostones, limestones, marls, and glauconitic shales with oyster beds, gastropods, echinoids, etc. It overlies conformably the Aptian–Albian Malha Formation and underlies the Cenomanian/Turonian shales of Abu Qada Formation. According to [47], the Cenomanian age of the Raha Formation is established from fossil evidence mainly echinoids and oysters. As suggested by [50], the Raha Formation was subdivided into a lower Abu Had Member and an upper Mellaha Sand Member. It is well exposed along the southern Tih scarp. It is replaced in northern Sinai by open-marine carbonate facies of the Halal Formation. On the west coast of the Gulf of Suez along the Galala scarps, [51] described a unit similar to the Raha Formation which is named the Galala Formation (Fig 2). According to [52], the Galala Formation of the north Eastern Desert is equivalent to the Raha and Abu Qada formations of southern Sinai.

#### 2.2.3. Bahariya Formation

[53] introduced the name Bahariya Formation for the Cenomanian deposits in the northern Western Desert. The type section of the Bahariya Formation occupies the floor and scarps of the oasis depression around Gebel Dist and has an exposed thickness of 170 m to more than 300 m underlying the El-Hefhuf Formation [40,54]. Lithologically, it is composed of sandstone, mudstone, and limestone. Its upper part is richer in shale than the lower one. The sandstones and mudstones are tan, greenish-grey, and glauconitic in parts, with many trace fossils [40] subdivided the Bahariya Formation into the lower Gebel Ghorabi Member, represented by fluviatile sandstone, and the upper Gebel Dist Member (estuarine-shallow marine), which is composed of intercalations of sandstones, siltstones, and claystones, partly glauconitic. It is strongly tidally influenced by some intercalations of lagoonal inputs. Some workers (e.g., [40]) treated the overlying El Heiz Formation as a third member, which comprises calcareous or siliceous sandstones and claystones that characterize a gradual regression of lagoonal and supratidal origin. In this work, the El Heiz Formation is considered a separate entity, following the widely accepted nomenclature of [53] and others. In the subsurface, the Bahariya Formation is widely distributed in the north Western Desert and represents oil-bearing units in different basins. It is of early Cenomanian age, based on foraminifera [55]. [40] concluded that the Bahariya Formation was deposited first under fluviatile, then under estuarine conditions, and finally under lagoonal conditions based entirely on lithological and palaeontological evidence.

The fossil content of the Bahariya Formation is highly diverse, including dinosaurs and other vertebrates along with invertebrates (e.g., [31,32,39–41,56]). Furthermore, it has also yielded abundant fossil plant remains (e.g., [11,12,16,57–61]). It is worth to be mentioned here that a stratigraphic unit that represents coastal mangrove deposits within the Bahariya Formation hosted an ecosystem characterized by a very high production of biomass, as evidenced by the very large dinosaurs such as *Paralititan*, fishes, and crocodiles among other fauna and flora [31].

The biota-rich horizons within the Bahariya Formation can be correlated, at least provisionally, with the Bahariya interval within the GPTSW-7 and other studied boreholes. This is supported stratigraphically by the fact that the Abu Roash G Member within the same wells represents the late Cenomanian episode, as demonstrated palynologically by [62].

#### 2.2.4. Maghrabi Formation

In the south Western Desert, the Bahariya Formation changes laterally into the Maghrabi Formation of [63], overlying unconformably the Sabaya Formation. In SW Egypt, the sedimentary succession of the Maghrabi Formation attains a maximum thickness of 180 m of grey claystone alternating with siltstones and sandstones. It contains fossil brachiopods (*Lingula* sp.), rare vertebrate remains (fish teeth, dinosaur bones, and turtle plates) and abundant plant remains (mainly angiosperm leaves), that even form thin coal beds near EI-Kharga [64]. [46] assumed mixed estuarine and tidal flat circumstances for the Maghrabi Formation in the surrounds of El-Kharga. They also dated the formation as late Cenomanian to early Turonian despite the lack of direct biostratigraphic evidence. This age determination is gained from correlation with the lateral counterpart defined as the ammonite-dated Galala Formation in the northeast direction.

Age determinations, based on palynology for the Maghrabi Formation and its equivalent in the Dakhla area, signify Albian to early Cenomanian age [65]. Triporate pollen, that are widely accepted as indicators of late Cenomanian or younger ages (e.g., [66,67]), have been reported from the Nubia Sandstone (in-part equivalent) in the Kharga Oasis area [68,69].

#### 2.2.5. Other equivalent localities

There are a few local exposures in Egypt also attributed to the Cenomanian that have so far received little attention and consequently few published data are available from such exposures. The palynological study of [27] focusing on the Umm Sidida Formation (Cenomanian) in the Gebel Abraq, southeast Aswan, represents a clear example of such equivalent rock units.

## 3. Material and methods

A data set consisting of a heterogeneous group of samples has been employed in the current study for palynological and macroflora investigations, comprising subsurface core and cuttings samples in addition to samples from surface exposures and specimens from older palaeontological collections (Table 1). Some of these samples were studied before by different authors under different aspects, and they are re-employed here to improve our interpretations (Table 1).

### 3.1. Megaflora

The studied material consists of fossil leaves, collected by members of the ‘Sonderforschungsbereich 69’ between 1981 and 1987 [70], now housed at the Museum für Naturkunde Berlin, Germany. This material was obtained from the Gebel El Dist, Naqb El Farafra, and Gebel El-Tibnia profiles representing the Bahariya Formation, as well as the Gebel El Minshera section and one profile from the Maghrabi Formation from Abu Tartur.

Concerning the Bahariya taxa, the first angiosperm-dominated assemblage comes from the Naqb El Farafra section and is preserved in sandstones corresponding to fluvial channels [40]. It comprises 29 specimens (Table 1). The second angiosperm-dominated assemblage comes from the Gebel El-Tibnia section and is preserved in clays corresponding to swamp sediments [40]. It encompasses only nine specimens (Table 1). The *Weichselia reticulata* and *Paradoxopteris stroemeri* specimens described by [60] from this collection apparently come from various localities, e.g., Gebel El Dist, South Bahariya, East Harra Escarpment.

The material from the Maghrabi Formation was collected from the Abu Tartur [71] in flaser-bedded sandstones in the basal part of the formation. It comprises 12 specimens (Table 1).

### 3.2. Palynoflora

**Fig 3:**
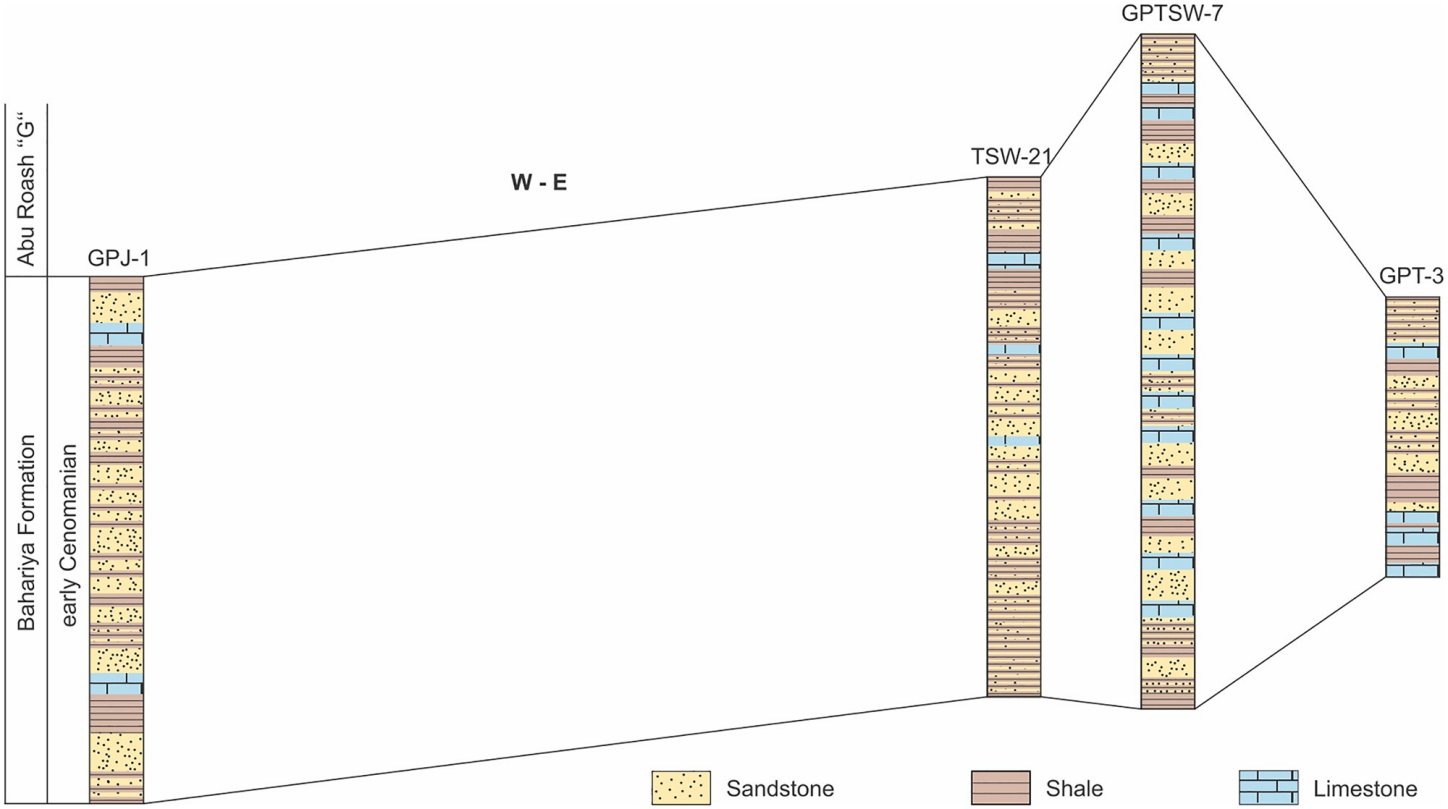
Lithostratigraphic logs of the Bahariya Formation in the studied wells in the north Western Desert, Egypt (based on [74–77]).

Fifty-six subsurface core and cuttings samples belonging to the early Cenomanian Bahariya Formation, gathered from four wells in the north Western Desert, Egypt, are palynologically investigated for this study. Such subsurface material is available, as the Bahariya Formation is an important target for petroleum exploration in the north Western Desert. The studied wells are GPJ-1, TSW-21, GPT-3, and GPTSW-7 wells (Fig 3). The Bahariya Formation in the GPTSW-7 and GPT-3 wells, was previously examined for its palynostratigraphy and palynofacies analysis ([62,72,73], respectively). However, the other two wells are investigated here for the first time and their results were supplemented with a re-interpretation of the data retrieved from both GPTSW-7 and GPT-3 boreholes, as outlined in Table 1.

Samples were processed following maceration techniques including cold hydrochloric and hydrofluoric acid (HCl-HF) demineralization but lacking alkali or oxidation handlings (e.g., [78]). The neutralized residues were sieved at 10 μm using a nylon mesh, stained with Safranin ‘O’, and strew-mounted on slides with the aid of Lucite International’s Elvacite 2044 acrylic resin. When drying, this offers a permanent mount with good optic properties, and notably for photography, the palynomorphs are all held to the surface of the coverslip and close to a single plane of focus. Relevant slides and residues are stored at the Geology Department, Faculty of Science, Mansoura University, Mansoura, Egypt.

### 3.3. Floristic composition and palaeoclimatic inferences

#### 3.3.1. Palynomorphs

**Fig 4:**
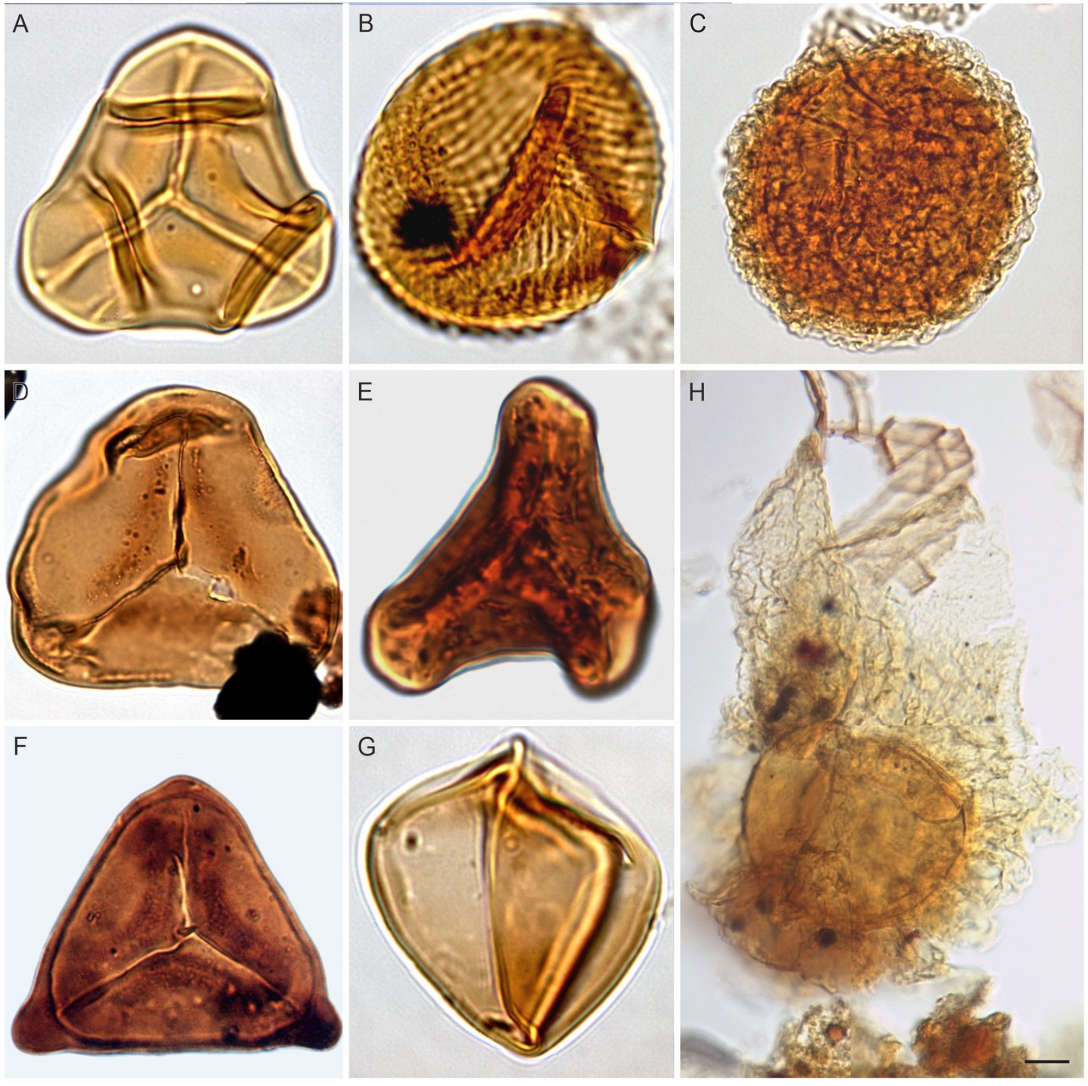
Spores from the Cenomanian of Egypt; The scale bar is equal to 10 μm. (A) *Cibotiumspora jurienensis* Filatoff 1975 (N. El-Faras 1X well), (B) *Cicatricosisporites minutaestriata* Bolkhovitina, 1961 (GPJ-1 well), (C) *Crybelosporites pannuceus* (Brenner) Srivastava, 1977 (N. El-Faras 1X well), (D) *Cyathidites australis* Couper, 1953 (GPJ-1 well), (E) *Concavisporites* sp. (GPJ-1 well), (F) *Trilobosporites laevigatus* El-Beialy, 1994 (Sharib-1X well; courtesy M. Zobaa), (G) *Triplanosporites* sp. (N. El-Faras 1X well), (H) *Ariadnaesporites* sp. (Faghur Hj5-1well).

**Fig 5:**
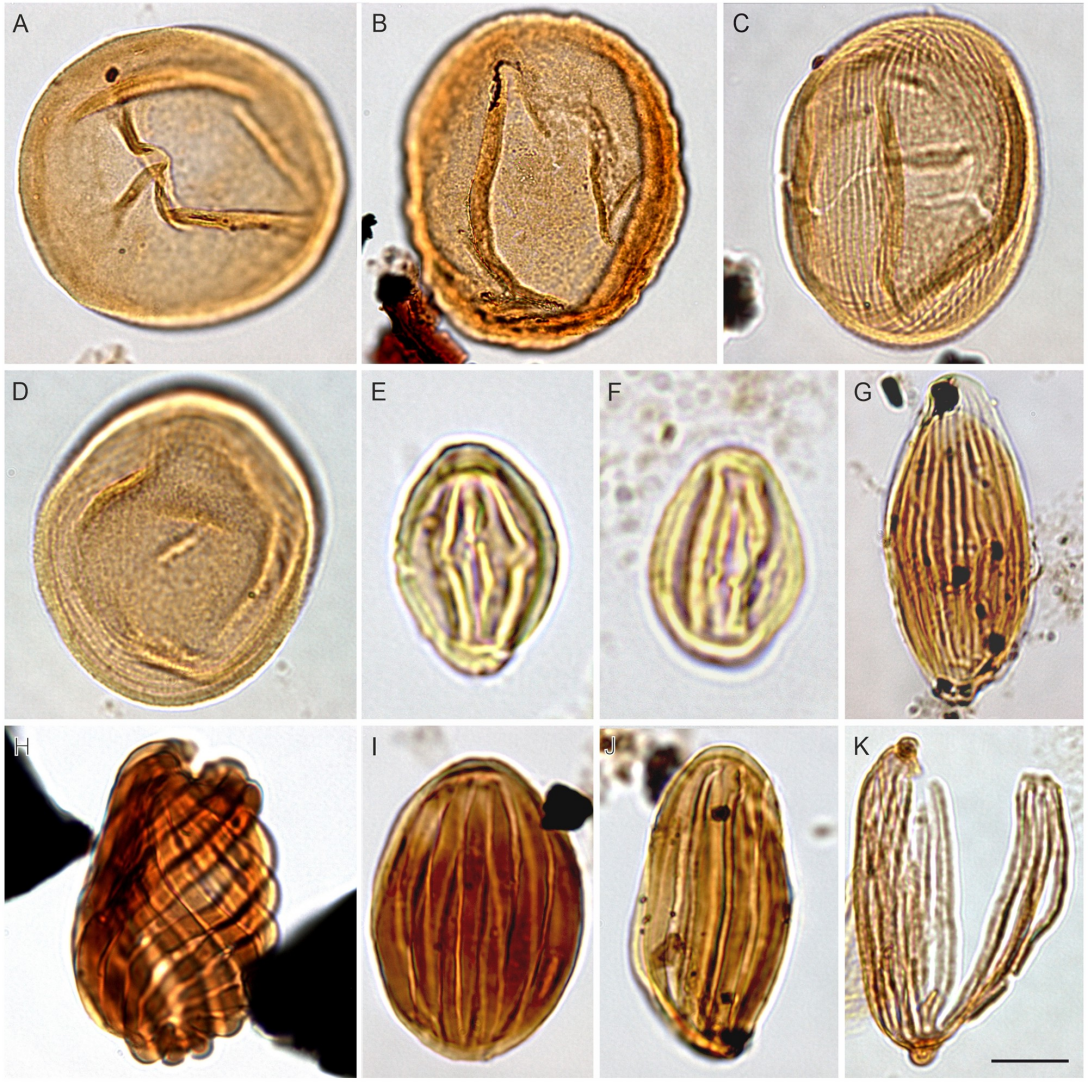
Gymnosperm pollen from the Cenomanian of Egypt; The scale bar is equal to 10 μm. (A) *Araucariacites australis* Cookson, 1947 (N. El-Faras 1X well), (B) *Balmeiopsis limbatus* (Balme) Archangelsky, 1977 (N. El-Faras 1X well), (C) *Classopollis brasiliensis* Herngreen, 1975 (N. El-Faras 1X well), (D) *Classopollis* sp. (N. El-Faras 1X well), (E) *Eucommiidites minor* Groot & Penny, 1961 (GPJ-1 well), (F) *Eucommiidites minor* Groot & Penny, 1961 (GPJ-1 well), (G) *Equisetosporites ambiguus* Hedlund, 1966 (GPT-3 well), (H) *Ephedripites jansonii* Muller, 1968 (GPJ-1 well), (I) *Ephedripites* sp. (GPT-3 well), (J) *Ephedripites* sp. (GPJ-1 well), (K) *Steevesipollenites* cf. *binodosus* Stover, 1964 (N. El-Faras 1X well).

**Fig 6:**
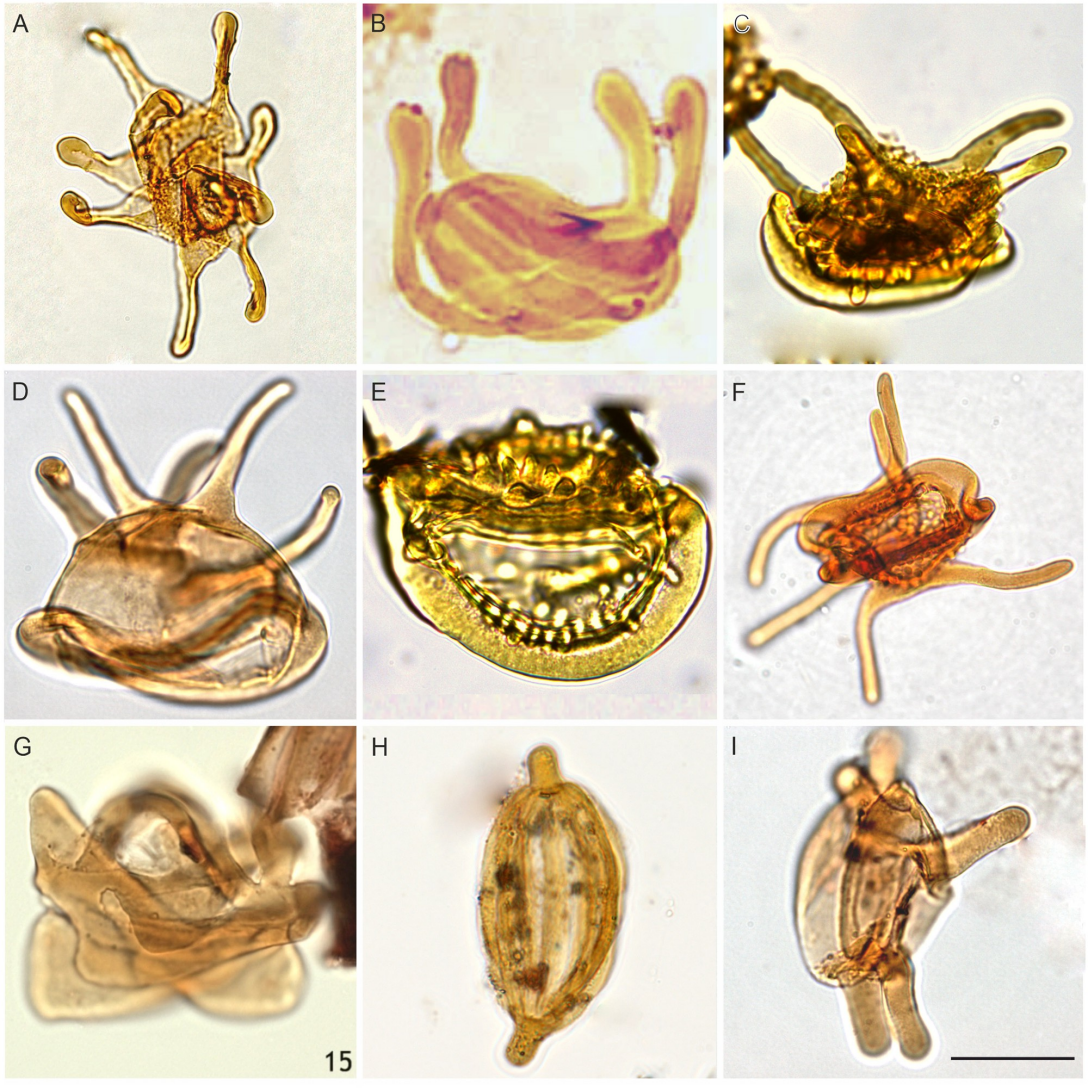
Elaterates from the Cenomanian of Egypt; The scale bar is equal to 10 μm. (A) *Elaterocolpites castelainii* Jardiné & Magloire, 1965 (GPJ-1 well), (B) *Elateroplicites africaensis* Herngreen, 1973 (GPTSW-7 well), (C) *Elaterosporites acuminatus* Jardiné, 1967 (Abu Tunis 1x well; courtesy A. Deaf), (D) *Elaterosporites klaszii* (Jardiné & Magloire) Jardiné, 1967 (N. El-Faras 1X well), (E) *Elaterosporites protensus* Jardiné, 1967 (Abu Tunis 1x well; courtesy A. Deaf), (F) *Elaterosporites verrucatus* Jardiné, 1967 (Razzak #7 well; courtesy M. Zobaa), (G) *Galeacorna causea* Stover, 1963 (Sharib-1X well; courtesy M. Zobaa), (H) *Senegalosporites petrobrasi* Herngreen, 1973 (GPTSW-7 well), (I) *Sofrepites legouxae* Jardiné, 1967 (N. El-Faras 1X well).

**Fig 7:**
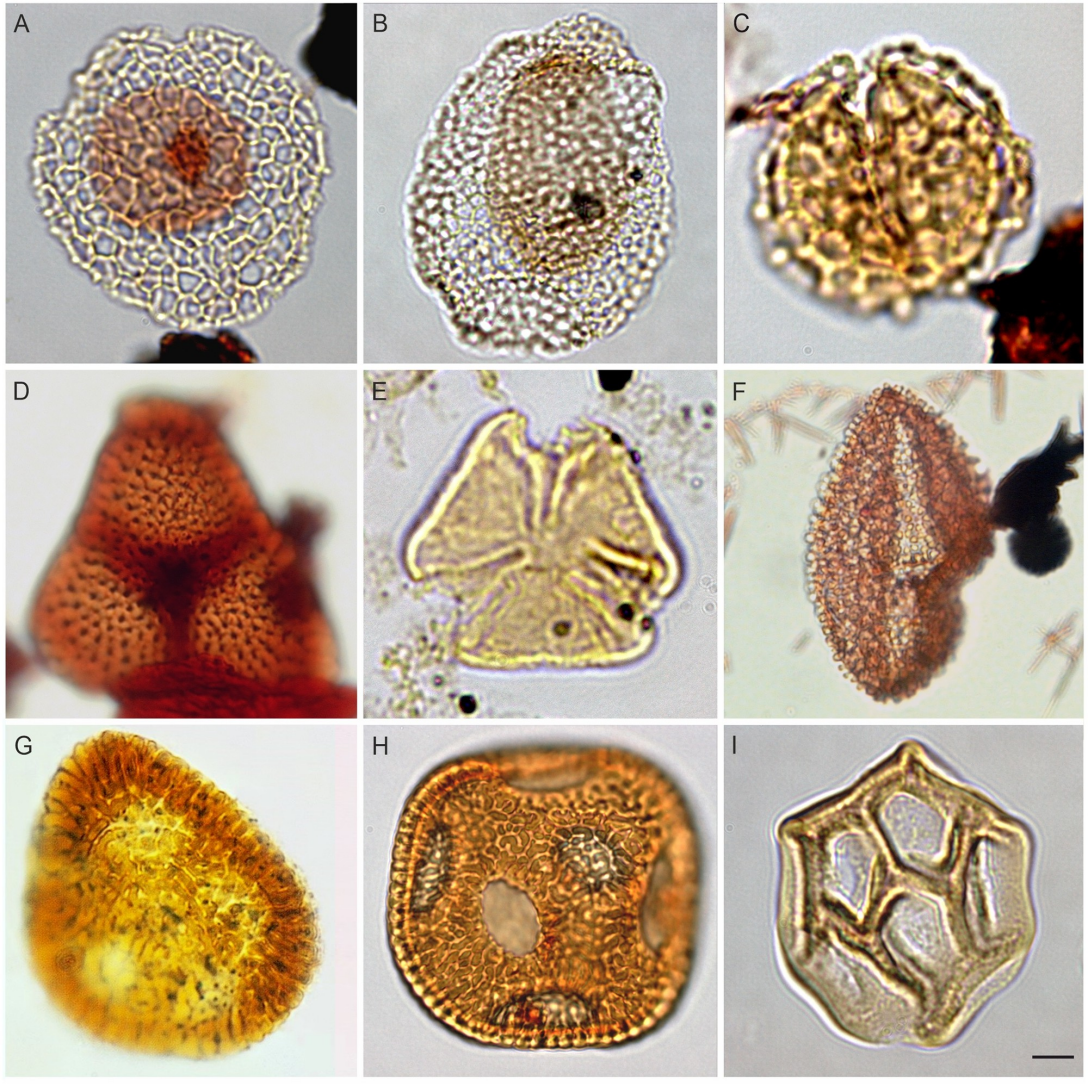
Pollen from the Cenomanian of Egypt; The scale bar is equal to 10 μm. (A) *Afropollis jardinus* (Brenner) Doyle, Jardiné & Doerenkamp, 1982 ((N. El-Faras 1X well), (B) *Afropollis kahramanensis* Ibrahim & Schrank, 1995 (N. El-Faras 1X well), (C) *Dichastopollenites ghazalatensis* Ibrahim, 1996 (N. El-Faras 1X well), (D) *Integritetradites porosus* Schrank & Mahmoud, 2000 (Faghur Hj5-1well), (E) *Nyssapollenites* sp. (GPJ-1 well), (F) *Stellatopollis* sp. (Faghur Hj5-1well), (G) *Cretacaeisporites aegyptiaca* Ibrahim, Zobaa, El-Noamani & Tahoun, 2017 (Lotus#3 well; courtesy M. Zobaa), (H) *Cretacaeisporites densimurus* Schrank and Ibrahim, 1995 (N. El-Faras 1X well), (I) *Cretacaeisporites polygonalis* (Jardiné & Magloire) Herngreen, 1974 (N. El-Faras 1X well).

For the purpose of this study, slides from each sample were (re-)examined and at least 200 sporomorphs (whenever possible) were counted (Table 2). Counts were accomplished in systematic transects to assure that no grains were lost. The rest of the slide was then examined in the same manner to identify rare taxa that were not present in the count data. Selected taxa are illustrated in Figs 4–7. All samples were analysed qualitatively (which palynomorphs are present in the individual samples) and quantitatively (how many specimens of each taxon are present in each sample). The authors are aware of the obvious shortcomings of such an approach, but a statistical analysis of the samples by means of cluster analysis, etc. provided no meaningful information, due to the relatively low number of palynomorphs that were present in most samples. Based on the qualitative composition of the samples, seven categories of palynomorphs, viz., algae, spores and pollen, dinoflagellate cysts, acritarchs, microforaminiferal test linings remains, fungal, and animal remains were considered.

**Table 2:** Relative abundances of palynomorphs recorded from the Bahariya Formation in the studied wells, north Western Desert, Egypt.

Well	Formation	Sample	Depth (m)	*Ariadnaesporites* spp.	*Cibotiumspora jurienensis*	*Cicatricosisporites minutaestriata*	*Cicatricosisporites* spp.	*Concavisporites* spp.	*Crybelosporites pannuceus*	*Cyathidites australis*	*Cyathidites minor*	*Gabonisporis vigourouxii*	*Leptolepidites* spp.	*Matonisporites* sp.	*Trilobosporites laevigatus*	*Triplanosporites* spp.	Indeterminate trilete forms	Total spores
GPJ-1	Bahariya	G1-52	1899		1			3	1	3	1					11		20
		G1-56	1935			1	2	1		3						3		10
		G1-60	1971				2	6	3	11						12		34
		G1-64	2007		1	5	17	8	7	4	1					9	2	54
		G1-68	2043			1	1	8	2	2						2	1	17
TSW-21		T21-3/3	2053					1		1						1		3
		T21-3/4	2054					1								5		6
		T21-3/5	2055				5	6	2	2	1					1		17
		T21-53	2016					2		5		3				8		18
		T21-56	2043				1	17	2	13	1					13		47
		T21-58	2061				3	6	1	12	4					14		40
		T21-60	2082	1			1	2		5	2					11		22
		T21-62	2100				3	3	2	6	1	1				7		23
		T21-64	2118				1	2	2	4	2					1		12
		T21-66	2136				1	15	5	6						5	2	34
		T21-68	2154		1		2	6	5	11	4			3		9	7	48
		T21-70	2172			3	9	19	7	8	1			4		17	3	71
		T21-72	2187					2	4	11	4			15		9	4	49
GPT-3		G3-79	2145					16	1	5	1					3		26
		G3-82	2172					4	1	7						3	3	18
		G3-85	2199							6	2					3	1	12
		G3-89	2235					1		3	1			1		2	2	10
GPTSW-7		G7-37	1914							18						8		26
		G7-38	1926	1		1				15				5				22
		G7-39	1938							10				1				11
		G7-40	1959					8		4				10		5		27
		G7-41	1962					7		5	7			5		6	1	31
		G7-42	1974							10	8		1	6		7	5	37
		G7-43	1986					1		12	5			8				26
		G7-44	1998			1	1	7		11				7		5	5	37
		G7-45	2007	1				1		5				6			1	14
		G7-46	2010						1	6	5			5			6	23
		G7-47	2021							12	1			1		5		19
		G7-48	2029							5				4		8	1	18
		G7-49	2031				1	1		7			1	3		7	7	27
		G7-50	2040					5	1	8			1	1		1	1	18
		G7-51	2052		1			15		6	5			6			5	38
		G7-52	2055		1	1		9		9			6	4		15	6	51
		G7-53	2058					1		10	5		1	11		5	5	38
		G7-54	2061					6		7	6		1			1	6	27
		G7-55	2064					5		9	8		1	5		4		32
		G7-56	2076					7		8	7			4		5	10	41
		G7-57	2079		1					6	8			5		1	4	25
		G7-58	2085	1						7	5			9		1	5	28
		G7-59	2088		1			1		9	8			7		5	2	33
		G7-60	2100					4		8	7		1	8		4	3	35
		G7-61	2103							6	6			6		1	4	23
		G7-62	2109			1		1		4	4		1	4		6		21
		G7-63	2112					5		5						4	4	18
		G7-64	2115							13	3			4		1	5	26
		G7-65	2124		8			7		21	2		1	5		1	3	48
		G7-66	2136					5		20	4			6		8	1	44
		G7-67	2139					1		15	3			9		7	2	37
		G7-68	2142					6		19	4			7		5	4	45
		G7-69	2151		1			7		17	7			5	1	6	3	47
		G7-70	2160							18	4		2	4		5	1	34
		G7-71	2172	3		3		7	5	20	5		7	3	15	20	8	96

Well	Formation	Sample	Depth (m)	*Araucariacites australis*	*Balmeiopsis limbatus*	*Classopollis brasiliensis*	*Classopollis* spp.	*Cycadopites carpentieri*	*Cycadopites nitidus*	*Cycadopites ovatus*	*Cycadopites* spp.	*Exesipollenites* sp.	*Monosulcites minimus*	*Monosulcites* spp.	Non-gnetalean gymnosperms
GPJ-1	Bahariya	G1-52	1899	1							1				2
		G1-56	1935	1							1				2
		G1-60	1971	2			5				4				11
		G1-64	2007	1							2				3
		G1-68	2043				1								1
TSW-21		T21-3/3	2053												0
		T21-3/4	2054												0
		T21-3/5	2055		1									1	2
		T21-53	2016	2			1				1				4
		T21-56	2043	4										2	6
		T21-58	2061	1	1		2							2	6
		T21-60	2082	1											1
		T21-62	2100	2		4					2				8
		T21-64	2118	1				1			1				3
		T21-66	2136	1		2	2				2			2	9
		T21-68	2154	5	2		8				2				17
		T21-70	2172	3	1		4				6				14
		T21-72	2187	5			4					1			10
GPT-3		G3-79	2145	9		3	15								27
		G3-82	2172	6			13								19
		G3-85	2199	10	4		27							1	42
		G3-89	2235	3			6								9
GPTSW-7		G7-37	1914			1									1
		G7-38	1926	1	1		5	1							8
		G7-39	1938	1			1								2
		G7-40	1959	8											8
		G7-41	1962												0
		G7-42	1974	8	1					1					10
		G7-43	1986	1	1				1	1					4
		G7-44	1998	1	1					1					3
		G7-45	2007		1										1
		G7-46	2010		7		6								13
		G7-47	2021												0
		G7-48	2029					1							1
		G7-49	2031	1	1		1			1					4
		G7-50	2040	1	1										2
		G7-51	2052		1										1
		G7-52	2055				1				1		1		3
		G7-53	2058												0
		G7-54	2061	1											1
		G7-55	2064												0
		G7-56	2076	1	10					5					16
		G7-57	2079		5				1	1					7
		G7-58	2085												0
		G7-59	2088	8	1		2								11
		G7-60	2100	8			2								10
		G7-61	2103	7											7
		G7-62	2109		1		11								12
		G7-63	2112	1			1				1				3
		G7-64	2115	1	22										23
		G7-65	2124	18	30		11	1			1		1		62
		G7-66	2136	8	8								1		17
		G7-67	2139	1	7		9								17
		G7-68	2142	17	9										26
		G7-69	2151	8	8		1	1							18
		G7-70	2160	1	1					1					3
		G7-71	2172	7			9				1				17

Well	Formation	Sample	Depth (m)	*Elaterocolpites castelainii*	*Elateroplicites africaensis*	*Elaterosporites klaszii*	*Ephedripites jansonii*	*Ephedripites* spp.	*Equisetosporites ambiguus*	*Eucommiidites minor*	*Eucommiidites troedssonii*	*Senegalosporites petrobrasi*	*Sofrepites legouxae*	*Steevesipollenites binodosus*	*Steevesipollenites* cf. *binodosus*	Gnetalean gymnosperms	Total gymnosperms
GPJ-1	Bahariya	G1-52	1899					1		2	1				4	8	10
		G1-56	1935					1		10	2					13	15
		G1-60	1971					4		42	13					79	90
		G1-64	2007	2	1	62		9		5						84	87
		G1-68	2043			2		1		4						9	10
TSW-21		T21-3/3	2053													0	0
		T21-3/4	2054													0	0
		T21-3/5	2055					4						2		9	11
		T21-53	2016					1							4	5	9
		T21-56	2043					6		6	1					21	27
		T21-58	2061				1	2		1						4	10
		T21-60	2082					10		2	1				1	15	16
		T21-62	2100						2	2	2			1		7	15
		T21-64	2118					3								8	11
		T21-66	2136					10		5	1					16	25
		T21-68	2154		1	2		7	1	5						16	33
		T21-70	2172			1		8		5						14	28
		T21-72	2187			4		10	1	2						17	27
GPT-3		G3-79	2145					2								2	29
		G3-82	2172							1						33	52
		G3-85	2199			1	1	2		1	2				1	8	50
		G3-89	2235					2		3						5	14
GPTSW-7		G7-37	1914													0	1
		G7-38	1926													0	8
		G7-39	1938													0	2
		G7-40	1959													0	8
		G7-41	1962					1								1	1
		G7-42	1974					1								1	11
		G7-43	1986													0	4
		G7-44	1998					1								1	4
		G7-45	2007	8		1		1								10	11
		G7-46	2010			8										8	21
		G7-47	2021	1		30										31	31
		G7-48	2029													0	1
		G7-49	2031	7	1	18										26	30
		G7-50	2040	2	3	45		1								51	53
		G7-51	2052	18		50										68	69
		G7-52	2055	8		18										26	29
		G7-53	2058	8		40		1								49	49
		G7-54	2061	1		18										19	20
		G7-55	2064	9		8										17	17
		G7-56	2076	1		40						1				42	58
		G7-57	2079	29		50		1								80	87
		G7-58	2085	8	1	8										17	17
		G7-59	2088	7		7		1								15	26
		G7-60	2100							1						1	11
		G7-61	2103	2		1		1								4	11
		G7-62	2109	8		7		1					1			17	29
		G7-63	2112	1												1	4
		G7-64	2115	1		1										2	25
		G7-65	2124							1						1	63
		G7-66	2136												1	19	36
		G7-67	2139					1					1			2	19
		G7-68	2142	1		1										2	28
		G7-69	2151	1						1						2	20
		G7-70	2160	2		1										3	6
		G7-71	2172	1	1	2				1					1	6	23

Well	Formation	Sample	Depth (m)	*Afropollis jardinus*	*Afropollis kahramanensis*	*Albertipollenites rosalindiae*	*Cretacaeisporites aegyptiaca*	*Cretacaeisporites densimurus*	*Cretacaeisporites polygonalis*	*Cretacaeisporites scabratus*	*Dichastopollenites dunveganensis*	*Dichastopollenites ghazalatensis*	*Foveomorphomonocolpites rashadi*	*Foveotricolpites giganteus*	*Foveotricolpites gigantoreticulatus*	*Integritetradites porosus*	*Monocolpopollenites* spp.	*Nyssapollenites* sp.	*Pennipollis peroreticulatus*	*Proteacidites* cf. *africaensis*
GPJ-1	Bahariya	G1-52	1899	5				1												
		G1-56	1935	25	1															
		G1-60	1971	48							2									
		G1-64	2007	69																
		G1-68	2043	12																
TSW-21		T21-3/3	2053	94													5			
		T21-3/4	2054	67										1			7			
		T21-3/5	2055	41	10												1			
		T21-53	2016	5	10			1						1						
		T21-56	2043	115	2												5			
		T21-58	2061	46	6						2						3		1	
		T21-60	2082	125	1					1	5						1	1		
		T21-62	2100	125	4									3			1	2		
		T21-64	2118	17				1		1							1			
		T21-66	2136	62					1	1									1	
		T21-68	2154	90	1			1		5				1				2	10	
		T21-70	2172	36		1		5											7	
		T21-72	2187	102	1			2	1	1									10	
GPT-3		G3-79	2145	1	1			1								1				
		G3-82	2172															1		
		G3-85	2199																	
		G3-89	2235																	
GPTSW-7		G7-37	1914	1	1															
		G7-38	1926	8	1															
		G7-39	1938		1										1					
		G7-40	1959	42																
		G7-41	1962	43																
		G7-42	1974	42											1					
		G7-43	1986	44																
		G7-44	1998	8																
		G7-45	2007	21	8															
		G7-46	2010	42	1											1				
		G7-47	2021	39																
		G7-48	2029	42																
		G7-49	2031	43	8															
		G7-50	2040	42	8															
		G7-51	2052	44																
		G7-52	2055	45																
		G7-53	2058	40																
		G7-54	2061	20																
		G7-55	2064	21	1															
		G7-56	2076	40																
		G7-57	2079	50								1								
		G7-58	2085	18																
		G7-59	2088	51	7							1								
		G7-60	2100	52								1								
		G7-61	2103	19																
		G7-62	2109	45			1					1								
		G7-63	2112	7																
		G7-64	2115	42		1														1
		G7-65	2124	43			8	18												
		G7-66	2136	42			1	8												
		G7-67	2139	44			1	42												
		G7-68	2142	42			1	40												
		G7-69	2151	43	1		8	18					1							
		G7-70	2160	30	6		1	1												
		G7-71	2172	50	8		1	19				1								

Well	Formation	Sample	Depth (m)	*Retimonocolpites variplicatus*Retimonocolpites spp. Retitricolpites sp. Retitricolporites pristinus Rousea sp. Stellatopollis sp. Syncolpites spp. Tetracolpites spp. Tricolpites spp. Tricolporopollenites spp. Total angiosperms Dinoflagellate cysts Acritarchs Microforaminiferal test linings Total palynomorph count Total sporomorphs count Spores % Gymnosperms % Non-gnetalean gymnosperms % Gnetalean gymnosperms % Angiosperms % Humidity indicators % Aridity indicators %
GPJ-1	Bahariya	G1-52	1899	1 1 8 4 42 38 47.6 23.8 4.8 19.0 19.0 47.6 11.9
		G1-56	1935	1 27 5 57 52 17.5 26.3 3.5 22.8 47.4 17.5 1.8
		G1-60	1971	1 51 2 175 175 19.4 51.4 6.3 45.1 29.1 19.4 5.1
		G1-64	2007	2 71 212 212 25.5 41.0 1.4 39.6 33.5 25.5 34.9
		G1-68	2043	1 13 1 40 40 42.5 25.0 2.5 22.5 32.5 42.5 10.0
TSW-21		T21-3/3	2053	1 1 101 104 104 2.9 0.0 0.0 0.0 97.1 2.9 0.0
		T21-3/4	2054	8 83 89 89 6.7 0.0 0.0 0.0 93.3 6.7 0.0
		T21-3/5	2055	1 1 2 56 1 84 84 20.2 13.1 2.4 10.7 66.7 20.2 7.1
		T21-53	2016	1 1 19 11 1 58 46 31.0 15.5 6.9 8.6 32.8 31.0 10.3
		T21-56	2043	1 3 126 5 205 200 22.9 13.2 2.9 10.2 61.5 22.9 2.9
		T21-58	2061	1 1 2 2 1 65 11 126 115 31.7 7.9 4.8 3.2 51.6 31.7 4.0
		T21-60	2082	1 135 27 1 1 201 173 10.9 8.0 0.5 7.5 67.2 10.9 5.5
		T21-62	2100	1 136 12 186 174 12.4 8.1 4.3 3.8 73.1 12.4 3.8
		T21-64	2118	1 21 44 44 27.3 25.0 6.8 18.2 47.7 27.3 6.8
		T21-66	2136	1 3 1 70 19 148 129 23.0 16.9 6.1 10.8 47.3 23.0 9.5
		T21-68	2154	6 1 117 2 1 200 198 24.0 16.5 8.5 8.0 58.5 24.0 9.5
		T21-70	2172	1 50 3 1 152 149 46.7 18.4 9.2 9.2 32.9 46.7 8.6
		T21-72	2187	1 3 121 3 2 200 197 24.5 13.5 5.0 8.5 60.5 24.5 9.5
GPT-3		G3-79	2145	1 5 24 4 84 60 31.0 34.5 32.1 2.4 6.0 31.0 23.8
		G3-82	2172	1 1 1 4 32 1 7 107 74 16.8 48.6 17.8 30.8 3.7 16.8 12.1
		G3-85	2199	1 2 3 12 5 77 65 15.6 64.9 54.5 10.4 3.9 15.6 41.6
		G3-89	2235	1 1 2 6 6 32 26 31.3 43.8 28.1 15.6 6.3 31.3 25.0
GPTSW-7		G7-37	1914	2 96 125 29 20.8 0.8 0.8 0.0 1.6 20.8 0.8
		G7-38	1926	8 17 53 100 47 22.0 8.0 8.0 0.0 17.0 22.0 5.0
		G7-39	1938	1 3 29 45 16 24.4 4.4 4.4 0.0 6.7 24.4 2.2
		G7-40	1959	1 1 44 20 1 99 79 27.3 8.1 8.1 0.0 44.4 27.3 0.0
		G7-41	1962	43 2 77 75 40.3 1.3 0.0 1.3 55.8 40.3 1.3
		G7-42	1974	8 1 2 54 9 111 102 33.3 9.9 9.0 0.9 48.6 33.3 0.9
		G7-43	1986	8 52 55 137 82 19.0 2.9 2.9 0.0 38.0 19.0 0.0
		G7-44	1998	1 1 10 90 141 51 26.2 2.8 2.1 0.7 7.1 26.2 0.7
		G7-45	2007	1 30 2 57 55 24.6 19.3 1.8 17.5 52.6 24.6 17.5
		G7-46	2010	1 2 47 69 160 91 14.4 13.1 8.1 5.0 29.4 14.4 8.8
		G7-47	2021	39 89 89 21.3 34.8 0.0 34.8 43.8 21.3 34.8
		G7-48	2029	42 22 83 61 21.7 1.2 1.2 0.0 50.6 21.7 0.0
		G7-49	2031	51 40 148 108 18.2 20.3 2.7 17.6 34.5 18.2 18.2
		G7-50	2040	1 51 61 183 122 9.8 29.0 1.1 27.9 27.9 9.8 27.9
		G7-51	2052	1 45 3 155 152 24.5 44.5 0.6 43.9 29.0 24.5 43.9
		G7-52	2055	1 46 1 127 126 40.2 22.8 2.4 20.5 36.2 40.2 21.3
		G7-53	2058	1 41 2 130 128 29.2 37.7 0.0 37.7 31.5 29.2 37.7
		G7-54	2061	20 29 96 67 28.1 20.8 1.0 19.8 20.8 28.1 19.8
		G7-55	2064	1 23 10 82 72 39.0 20.7 0.0 20.7 28.0 39.0 20.7
		G7-56	2076	1 41 1 141 140 29.1 41.1 11.3 29.8 29.1 29.1 29.8
		G7-57	2079	1 52 40 204 164 12.3 42.6 3.4 39.2 25.5 12.3 39.2
		G7-58	2085	18 17 80 63 35.0 21.3 0.0 21.3 22.5 35.0 21.3
		G7-59	2088	59 3 121 118 27.3 21.5 9.1 12.4 48.8 27.3 14.0
		G7-60	2100	53 3 102 99 34.3 10.8 9.8 1.0 52.0 34.3 2.0
		G7-61	2103	19 9 62 53 37.1 17.7 11.3 6.5 30.6 37.1 6.5
		G7-62	2109	1 1 49 1 100 99 21.0 29.0 12.0 17.0 49.0 21.0 28.0
		G7-63	2112	7 29 29 62.1 13.8 10.3 3.4 24.1 62.1 6.9
		G7-64	2115	44 1 96 95 27.1 26.0 24.0 2.1 45.8 27.1 2.1
		G7-65	2124	1 1 71 18 200 182 24.0 31.5 31.0 0.5 35.5 24.0 5.5
		G7-66	2136	8 59 12 151 139 29.1 23.8 11.3 12.6 39.1 29.1 0.7
		G7-67	2139	1 88 144 144 25.7 13.2 11.8 1.4 61.1 25.7 7.6
		G7-68	2142	1 84 8 165 157 27.3 17.0 15.8 1.2 50.9 27.3 1.2
		G7-69	2151	8 79 9 155 146 30.3 12.9 11.6 1.3 51.0 30.3 1.3
		G7-70	2160	1 39 8 87 79 39.1 6.9 3.4 3.4 44.8 39.1 3.4
		G7-71	2172	79 20 218 198 44.0 10.6 7.8 2.8 36.2 44.0 6.4

Thereafter, the occurrences of relevant sporomorphs were used for the palaeoenvironmental reconstruction in the sense of the Sporomorph Ecogroup (SEG) model of [79,80], which denotes the total types of dispersed spores and pollen of land plants that reflect the composition of an individual source community (Table 3). Additionally, palynomorphs were categorized into four systematic groups to enable the description of the floristic composition: spores, gymnosperm (non-gnetalean and gnetalean), and angiosperm pollen [81]. Sporomorphs have been grouped, based on their own botanical affinities to study large-scale vegetation variation in the Bahariya Formation and its equivalents (Table 3). The floristic composition of the Bahariya Formation in the studied wells was determined from palynomorph abundance. In addition, humid-indicating vs. arid-indicating sporomorphs relationships are also highlighted (e.g., [81–84]). In this study, we follow the approach of [81], who deduced the degree of aridity vs. humidity by using indicator taxa that have been identified to favour a specified environment based on known climatic preferences of their extant relatives (e.g., *Ephedra*). Cretaceous indicators of arid climates include *Classopollis*, ephedroid pollen grains, and elater-bearing species, while the Cretaceous indicators of humid climates comprise fern spores. The abundance of dry and humid indicators was determined for each sample to find out changes in the climatic conditions across the studied sequences (Table 4).

**Table 3:** List of the recorded taxa (arranged alphabetically) from the Cenomanian of Egypt and their botanical affinities. SEG classification, compiled after [79].

Species	Botanical affinity	SEG	Remarks
**I: Algae**			
*Botryococcus* cf. *B*. *braunii*	Botryococcaceae [85]		
*Pediastrum* spp.	Hydrodictyaceae [85]		
*Schizosporis reticulatus*	Unknown chlorophycean family [85]		
**II. Spores**:			
Ferns:			
*Ariadnaesporites* spp.	Salviniaceae [86]		
*Cibotiumspora jurienensis*	Filicopsida [87]		
*Cicatricosisporites minutaestriata*	Schizaeaceae, *Anemia* [88]	Lowlands	
*Cicatricosisporites* spp.	Schizaeaceae, Anemeaceae [88]	Lowlands	
*Concavisporites* spp.	Matoniaceae [89]	Lowlands	
*Crybelosporites pannuceus*	Marsileaceae [90]		
*Cyathidites australis*	Filicopsida: various genera and families; Cyatheaceae, *Cyathea*, Dicksoniaceae, Schizaeaceae, *Lygodium* [87]		
*Cyathidites minor*	Filicopsida: many different families and genera e.g., Cyatheaceae (*Cyathea*), Dicksoniaceae, Schizaeaceae (*Lygodium*); similar spores isolated from fossils of *Coniopteris hymenophylloides*, *Eboracia lobifolia*, *Dicksonia mariopteris*, *Thyrsopteris elegans* [87]		
*Gabonisporis vigourouxii*	Marsileaceae [90]		
*Leptolepidites* spp.	Tree ferns [91]	River	
*Matonisporites* spp.	Matoniaceae [91]	Lowlands	
*Trilobosporites laevigatus*	Matoniaceae	Lowlands	
*Triplanosporites* spp.	Lygodiaceae, *Lygodium* [92]		More than 40 species of *Lygodium* are distributed in the tropical and subtropical regions of America, Asia, Australia, and Africa [92]
**III. Gymnosperms**:			
*Araucariacites australis*	Conifers: Araucariaceae [93], *Araucaria*, *Agathis* [87]	Coastal	*Araucaria* and *Agathis* are genera of evergreen coniferous trees in the family Araucariaceae. There are 19 extant species in the genus *Araucaria* in New Caledonia (where 13 species are endemic), Norfolk Island, eastern Australia, New Guinea, Argentina, Chile, and southern Brazil [94], as well as 19 extant species in the genus *Agathis* in Peninsular Malaysia to New Zealand, including Malesia, the Philippines, New Guinea, Melanesia and Australia [95]
*Balmeiopsis limbatus*	Conifers: Araucariaceae [96]		*Balmeiopsis* type spores were recovered from the male cone of *Brachyphyllum irregulare* [97]
*Classopollis brasiliensis*	Conifers: Cheirolepidiaceae [98]	Coastal	
*Classopollis* spp.	‘‘	Coastal	
*Cycadopites carpentieri*	Cycadales, Ginkgoales, Peltaspermales and Benettitales [99]	Lowlands	*Cycadopites*-like pollen was also obtained from Pentoxylaceae and Peltaspermales [98]
*Cycadopites nitidus*	‘‘	Lowlands	
*Cycadopites ovatus*	‘‘	Lowlands	
*Cycadopites* spp.	‘‘		
*Ephedripites jansonii*	Gnetales: Ephedraceae, *Ephedra* [98]		*Ephedra* is mostly a xeromorphic genus in the northern hemisphere in central Europe, western Mediterranean, central Asia, North Africa, and North America [100,101]
*Ephedripites* spp.	‘‘		
*Eucommiidites minor*	Erdtmanithecales: Erdtmanithecaceae [102]		
*Eucommiidites troedssonii*	‘‘	Drier Lowlands	
*Equisetosporites ambiguus*	Gnetales: Ephedraceae, *Ephedra* [98]		
* *Equisetosporites lawalii*	‘‘		
*Exesipollenites* sp.	Cycadophytes, Bennettitales [103]	Drier Lowlands	*Exesipollenites* is produced by some bennettitalean plants such as *Nilssoniopteris* and indicates a dry microclimate influenced by salty wind [103]
* *Gnetaceaepollenites* spp.	Gnetales: Ephedraceae, *Ephedra* [98]		
*Monosulcites minimus*	Ginkgoales [93]	Lowlands	
*Monosulcites* spp.	Cycadales, Bennettitales [93]	Lowlands	
*Steevesipollenites binodosus*	Gnetales: Ephedraceae, *Ephedra* [98]		
*Steevesipollenites* cf. *binodosus*	‘‘		
Elaterate Complex:			
*Elaterocolpites castelainii*	Gnetales		
*Elateroplicites africaensis*	‘‘		
* *Elaterosporites acuminatus*	‘‘		
*Elaterosporites klaszii*	‘‘		
* *Elaterosporites protensus*	‘‘		
* *Elaterosporites verrucatus*	‘‘		
* *Galeacornea causea*	‘‘		
* *Galeacornea clavis*	‘‘		
*Senegalosporites petrobrasi*	‘‘		
*Sofrepites legouxae*	‘‘		
**IV. Angiosperms**:			
*Afropollis jardinus*	*Afropollis* might represent an unknown group of seed plants and not necessarily an angiosperm [1]		
*Afropollis kahramanensis*	‘‘		
*Albertipollenites rosalindiae*	[104] assumed that its *Dolichandrone* type belongs to Bignoniaceae. Affinity to Dipterocarpaceae is presumed by [105], whereas other affinities could not be excluded		
*Cretacaeisporites aegyptiaca*	Dicotyledon: Periporate pollen forms are produced by extant families such as the Chenopodiaceae, Amaranthaceae, and Caryophyllaceae		
*Cretacaeisporites densimurus*	‘‘		
*Cretacaeisporites polygonalis*	‘‘		
*Cretacaeisporites scabratus*	[106] suggested an affinity with the genera *Trimenia*, *Thalictrum*, and *Alisma*. [107] also noted the similarity of some species of *Cretacaeiporites* with pollen of the family Caryophyllaceae. *C*. *scabratus* pollen from the Cretaceous (probably late Cenomanian) of Gabon shows strong similarity with pollen of some extant Ranunculaceae (e.g., *Anemone*, *Coptis*, *Hepatica*), as outlined by [108]		[1] considered the presence of *C*. *scabratus* in the Albian-Cenomanian of Brazil, as the oldest potential record of the Trimenaceae. However, [108], have compared several ultrastructural features of *C*. *scabratus* with the Ranunculaceae, and suggest that some species of the genus derive from probable tricolpate ancestors
*Dichastopollenites dunveganensis*	Monocotyledon, “magnoliid” affinity [109]		
*Dichastopollenites ghazalatensis*	‘‘		
*Foveomorphomonocolpites rashadi*	Monocotyledon, “magnoliid” affinity		
*Foveotricolpites giganteus*	Dicotyledon		
*Foveotricolpites gigantoreticulatus*	Dicotyledon		
*Integritetradites porosus*	Dicotyledon		
*Monocolpopollenites* spp.	Dicotyledon		
*Nyssapollenites* sp.	Some species of *Nyssapollenites* have similar morphology to the pollen of the extant families Nyssaceae, Araliaceae, Cornaceae, and Marcgraviaceae (see [110])		
*Pennipollis peroreticulatus*	Monocotyledon, Alismatales [111]		
*Proteacidites* cf. *africaensis*	Dicotyledon		
* *Proxapertites* sp.	Araceae [112]		
*Retimonocolpites variplicatus*	Monocotyledon, “magnoliid” affinity [109]		
*Retimonocolpites* spp.	‘‘		
*Retitricolpites* sp.	Dicotyledon		
*Retitricolporites pristinus*	‘‘		
* *Rhoipites* spp.	‘‘		
*Rousea* sp.	Unknown, although [113] compared his type spices *Rousea subtlis* with the Family Salicaceae. Moreover, [104] assumed that its *Dolichandrone* type belongs to Bignoniaceae		
*Stellatopollis* sp.	Monocotyledon, “magnoliid” affinity [114]		
* *Striatopollis* spp.			
*Syncolpites* spp.	Dicotyledon		
*Tetracolpites* spp.	Dicotyledon		
*Tricolpites* spp.	Magnoliopsida: Ericales, Hamamelidales, Saxifragales [98]		
*Tricolporopollenites* spp.	Dicotyledon		
**V. Incertae sedis**:			
* *Reyrea polymorpha*			

* represents taxa that are not recorded in the studied boreholes but reported earlier in Egyptian strata.

**Table 4:** Quantitative data for the floristic composition and palaeoclimatic implications of the studied boreholes, north Western Desert, Egypt.

Variable	GPJ-1	TSW-21	GPT-3	GPTSW-7
Average total palynomorph count	99.0	137.0	67.0	123.0
Number of samples (counts >100)	2.0	9.0	0.0	22.0
Average angiosperm abundance	33.9	61.4	0.0	35.6
Average non-gnetalean gymnosperm abundance	3.7	4.4	33.1	6.8
Average gnetales abundance	29.8	7.6	14.8	11.5
Average spores abundance	30.5	21.9	23.7	28.3
Average humidity indicators	30.5	21.9	23.7	28.3
Average aridity indicators	12.7	6.0	25.6	12.5

#### 3.3.2. Megaflora

Although the main aim of this work is beyond taxonomy, a preliminary taxonomical revision of the Berlin material (consisting solely of leaf impressions) was done to allow for comparison with other floras. We used the nomenclature of the Manual of Leaf Architecture of [115] for the description of leaves, to be able to disentangle the systematics of the specimens for comparison with the original determinations of [58–60]. Other specimens curated in other collections are beyond the focus of this study.

No quantitative climate analyses based on leaf physiognomy such as CLAMP were done because of the low number of morphotypes, which could be due to the poor sampling effort or the taphonomical bias. Nevertheless, a preliminary estimate of the annual rainfall was calculated, based on the correlation with leaf size, using the regression formula provided by [116], but it should be clear that this estimation has to be interpreted with great care.

## 4. Results

### 4.1. Megafloral results

**Fig 8:**
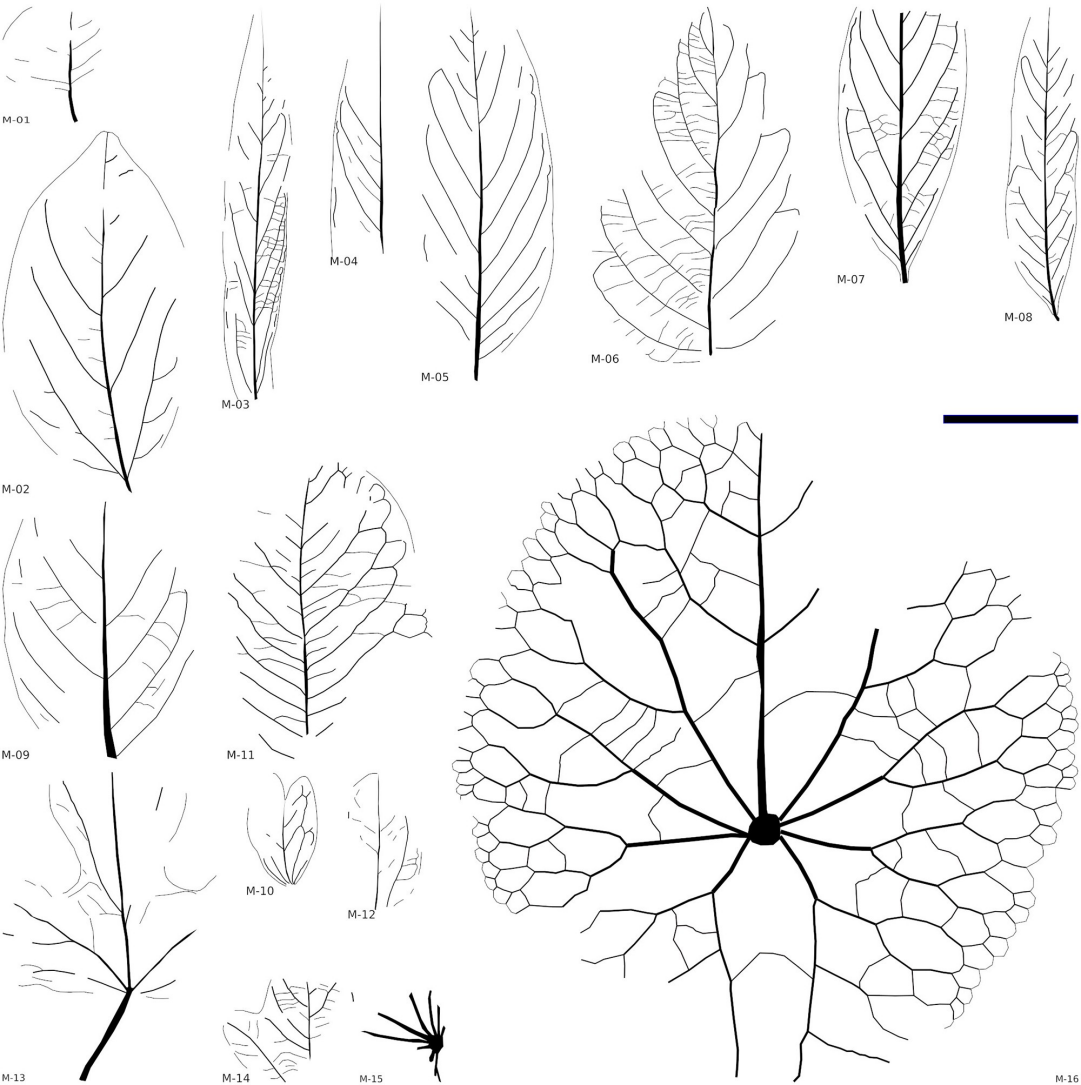
Line drawings of plant macro-remains from the Cenomanian of Egypt. The scale bar is equal to 5 cm. (M-01) Morphotype-01 (Gebel El-Tibnia, MB.Pb.2022/1019), (M-02) Morphotype-02 (Naqb El Farafra, B0067), (M-03) Morphotype-03 (Naqb El Farafra, B0012), (M-04) Morphotype-04 (Gebel El-Tibnia, MB.Pb.2022/1034), (M-05) Morphotype-05 (Naqb El Farafra, B0007), (M-06) Morphotype-06 (Naqb El Farafra, B0005), (M-07) Morphotype-07 (Naqb El Farafra, B0010), (M-08) Morphotype-08 (Naqb El Farafra, B0009), (M-09) Morphotype-09 (Naqb El Farafra, B0115), (M-10) Morphotype-10 (Naqb El Farafra, B0116), (M-11) Morphotype-11 (Naqb El Farafra, B0070), (M-12) Morphotype-12 (Gebel El-Tibnia, MB.Pb.2022/1044), (M-13) Morphotype-13 (Gebel El-Tibnia, B0006), (M-14) Morphotype-14 (Gebel El-Tibnia, B0110), (M-15) Morphotype-15 (Gebel El-Tibnia, B0023), (M-16) Morphotype-16 (Naqb El Farafra, B0001).

Sixteen morphotypes of dicots, two monocots, and one fern were recorded from the Cenomanian material considered here (Fig 8). These were identified from three microfacies assemblages representing the Bahariya Formation and its Maghrabi Formation equivalent: sandstones with dicots, claystones with angiosperms, especially aquatics and monocots, and siltstones with *Weichselia reticulata*.

Leaf Morphotypes (Bahariya and Maghrabi):

Non-monocot angiosperm morphotypes are here presented within a dichotomous key highlighting the most salient features distinguishing them.

1. leaves simple, pinnately veined, entire margined leaves.

1.1. secondary venation poorly visible. Lamina ovate, L:W >2, microphyllous:

**Morphotype-1**

1.2. secondary venation eucamptodromous.

1.2.1. one pair of acute basal secondaries, agrophic veins simple, minor secondaries eucamptodromous. Higher-order venation poorly visible:

**Morphotype-02**

1.2.2. angle of secondaries uniform.

1.2.2.1 less than one intersecondary per intercostal area:

**Morphotype-03**

1.2.2.2 one intersecondary per intercostal area:

**Morphotype-04**

1.2.3. angle of secondaries smoothly increasing proximally.

1.2.3.1 higher-order venation is poorly visible:

**Morphotype-05**

1.2.3.2. tertiaries prominent, percurrent. Tertiary angle increasing proximally, being basally obtuse and apically acute to the mid-vein:

**Morphotype-06**

1.3. secondary venation eucamptodromous becoming brochidodromous distally:

**Morphotype-07**

1.4. secondary venation simple brochidodromous:

1.4.1 apex acute:

1.4.1.1 Leaf shape very elongate (L:W>5), ovate, notophyllous, apex acute, base acute and convex. Intersecondaries absent, tertiary venation mixed opposite/alternate percurrent, obtuse to the mid-veins:

**Morphotype-08**

1.4.1.2 leaf shape broadly elliptic (L:W ca 2), mesophyllous:

**Morphotype-09**

1.4.2 apex emarginate:

**Morphotype-10**

1.5. secondary venation festooned brochidodromous, more than one intersecondary per intercostal area, tertiaries opposite percurrent, slightly acute to the midvein. Leaf shape unknown but at least mesophyllous, apex obtuse, base unknown:

**Morphotype-11**

2. primary venation acrodromous:

**Morphotype-12**

3. primary venation palmate actinodromous.

3.1.1. petiole marginal, lamina lobed, apex unknown, base cordate, mesophyllous; secondary venation unknown, agrophic veins simple, higher-order venation unknown:

**Morphotype-13**

3.1.2. A highly incomplete leaf differs from morphotype 11 by the shape of the lobes: in morphotype 11, the lobe has an enlarged apical part, while in this morphotype, lobes are very short and rounded:

**Morphotype-14**

3.2. leaf attachment peltate, lamina unlobed.

3.2.1. not distinct mid-vein, at least 10 primaries:

**Morphotype-15**

3.2.2. distinct midvein, 9 primaries; secondaries probably festooned brochidodromous; tertiaries opposite percurrent, convex, at least mesophyllous, differs also from morphotype 12 by the strong relief of the venation:

**Morphotype-16**

**Morphotype-01**

**Fig 9:**
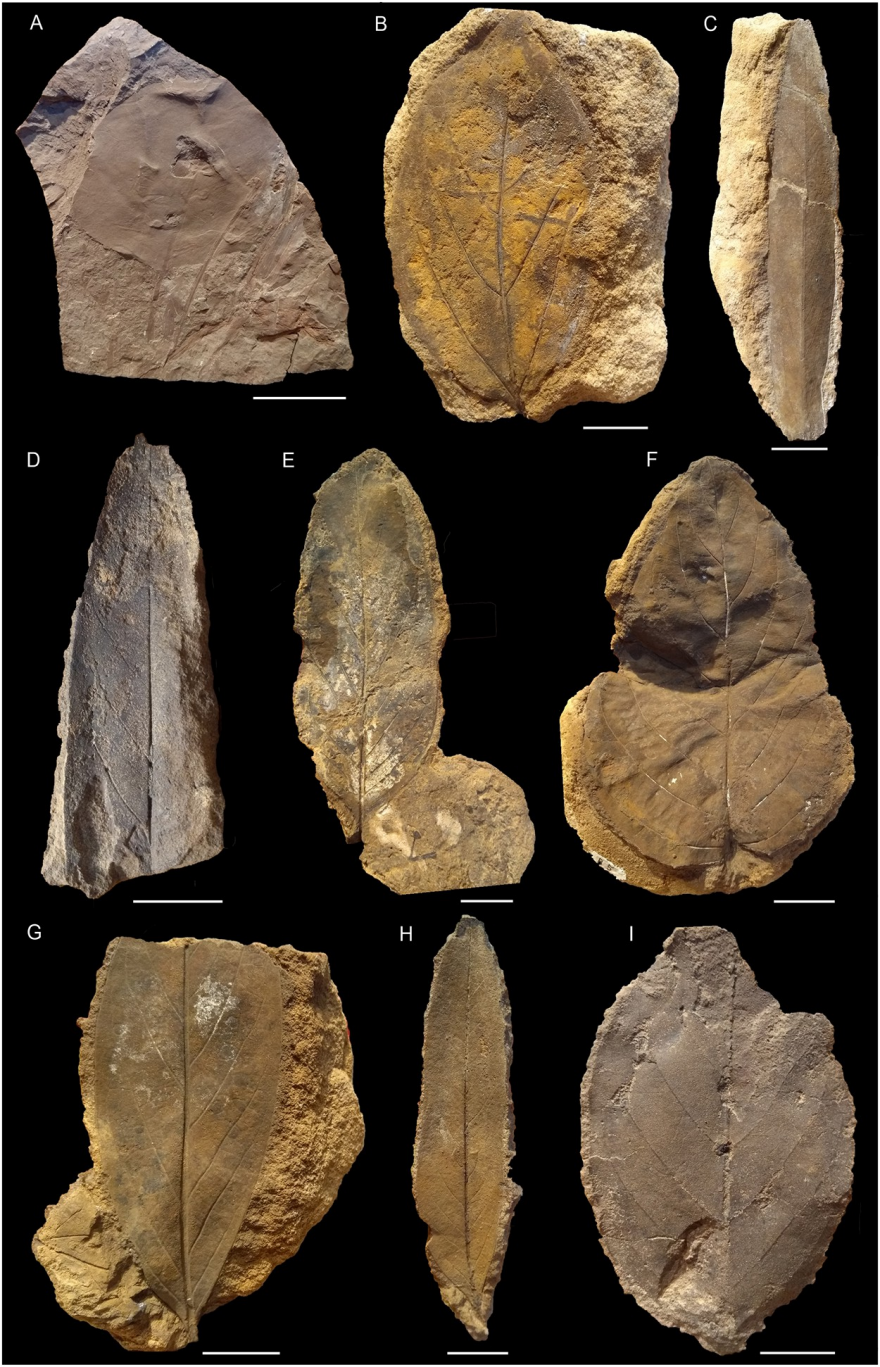
Plant macro-remains from the Cenomanian of Egypt; The scale bar is equal to 2 cm. (A) Morphotype-01 (Gebel El-Tibnia, MB.Pb.2022/1019), (B) Morphotype-02 (Naqb El Farafra, B0067), (C) Morphotype-03 (Naqb El Farafra, B0012), (D) Morphotype-04 (Gebel El-Tibnia, MB.Pb.2022/1034), (E) Morphotype-05 (Naqb El Farafra, B0007), (F) Morphotype-06 (Naqb El Farafra, B0005), (G) Morphotype-07 (Naqb El Farafra, B0010), (H) Morphotype-08 (Naqb El Farafra, B0009), (I) Morphotype-09 (Naqb El Farafra, B0115).

Material: one specimen (Figs 8 and 9A) from the Gebel El-Tibnia section (Bahariya) and another specimen from Abu Tartur (Maghrabi).

Affinity: unknown, the specimen from the Abu Tartur may belong to another morphotype.

Description: leaves petiolate and simple; petiolar attachment marginal; lamina microphyllous, L:W ratio <2; lamina ovate, symmetrical, unlobed and untoothed, chartaceous; apex and base obtuse; primary venation pinnate, higher-order venation unknown.

**Morphotype-02**

Material: specimen B0067 (Figs 8 and 9B) from Naqb El Farafra section (*Magnoliaephyllum auriculatum* according to [60]) and specimens B1078 as well as MB.Pb.2022/1046 and MB.Pb.2022/1047 from Abu Tartur.

Affinity: possibly lauraceous.

Description: leaves simple; petiolar attachment marginal; lamina mesophyllous, L:W ratio about 2; lamina ovate, symmetrical, unlobed and untoothed, coriaceous; apex acute and convex, base obtuse; primary venation pinnate, agrophic veins simple, secondary venation eucamptodromous, interior secondaries absent, one pair of acute basal secondaries, higher-order of venation unknown.

**Morphotype-03**

Material: specimens B0011, B0012 from Naqb El Farafra (Figs 8 and 9C; *Rogersia longifolia* Fontaine, according to [60]).

Affinity: possibly lobes of a much bigger leaf of *Sapindopsis* type.

Description: leaves simple; petiolar attachment unknown; lamina noto- to mesophyllous, L:W ratio > 5; lamina elliptic, symmetrical, unlobed and untoothed, coriaceous; apex acute, base unknown; primary venation pinnate, agrophic veins absent, secondary venation eucamptodromous, interior secondaries absent, angle of secondaries uniform, less than one intersecondary per intercostal area, tertiaries percurrent, obtuse to the midveins, higher-order of venation unknown.

**Morphotype-04**

Material: only specimen MB.Pb.2022/1034 from the Gebel El-Tibnia section (Figs 8 and 9D).

Affinity: unknown.

Description: leaves simple; petiolar attachment unknown; lamina at least notophyllous, L:W ratio > 2; lamina shape unknown but unlobed and untoothed, chartaceous; apex acute, base unknown; primary venation pinnate, secondary venation eucamptodromous, angle of secondaries uniform, one intersecondary per intercostal area, higher order of venation unknown.

**Morphotype-05** (Figs 8 and 9E; *Laurophyllum africanum* Lejal-Nicol et Dominik).

Material: specimens B0007 (*Magnoliaephyllum bahariyense*) and B0020 (*Laurophyllum africanum*) from Naqb El Farafra.

Affinity: unknown, specimens of this morphotype represent the holotypes of *Magnoliaephyllum bahariyense* Lejal-Nicol et Dominik (B0007) and *Laurophyllum africanum* Lejal-Nicol et Dominik (B0020).

Description: leaves simple; petiolar attachment marginal; lamina micro- to mesophyllous, L:W ratio 2–5; lamina ovate, symmetrical, unlobed and untoothed, coriaceous; apex unknown, base acute, cuneate; primary venation pinnate, agrophic veins absent, secondary venation eucamptodromous, interior secondaries absent, angle of secondaries smoothly increasing proximally, less than one intersecondary per intercostal area, higher order of venation unknown.

**Morphotype-06** (*Cornophyllum distense* Lejal-Nicol et Dominik).

Material: specimens B0005 (Figs 8 and 9F; *Cornophyllum distense*), B0008 (*Cornophyllum* sp.), and B0069a (*Magnoliaephyllum auriculatum*) from Naqb El Farafra.

Affinity: highly organized tertiary venation, possibly a eudicot. This morphotype represents the holotype of *Cornophyllum distense* Lejal-Nicol et Dominik (B5).

Description: leaves simple; petiolar attachment marginal; lamina mesophyllous, L:W ratio ca 2; lamina ovate, symmetrical, unlobed and untoothed, coriaceous; apex acute, base obtuse, rounded; primary venation pinnate, agrophic veins absent, secondary venation eucamptodromous, interior secondaries absent, angle of secondaries smoothly increasing proximally, less than one intersecondary per intercostal area, tertiaries prominent, percurrent, tertiary angle increasing proximally, being basally obtuse and apically acute to the midvein, higher order of venation unknown.

**Morphotype-07**

Material: specimens B0010 (Figs 8 and 9G; *Laurophyllum africanum*), B0068 (*Magnoliaephyllum bahariyense*), B0109 (*L*. *africanum*) and B155 (*Cornophyllum venustum*) from Naqb Farafra as well as specimens B1434, B1435 and MB.Pb.2022/1048 from Abu Tartur.

Affinity: unknown (lauroid?). This morphotype represents a paratype of *M*. *bahariense* Lejal-Nicol et Dominik (B68).

Description: leaves simple; petiolar attachment marginal; lamina mesophyllous, L:W ratio 4–5; lamina ovate, symmetrical, unlobed and untoothed, coriaceous; apex acute, acuminate, base acute, convex; primary venation pinnate, agrophic veins absent, secondary venation eucamptodromous becoming brochidodromous distally, interior secondaries absent, angle of secondaries smoothly decreasing proximally, less than one intersecondary per intercostal area, tertiary venation mixed opposite/alternate percurrent, obtuse to the midvein, higher order of venation unknown.

**Morphotype-08**

Material: specimens B0072 (*Rogersia angustifolia*), B0009 (Figs 8 and 9H; *Laurophyllum lanceolatum*), MB.Pb.2022/0915, MB.Pb.2022/0942, MB.Pb.2022/0970, MB.Pb.2022/0971 and MB.Pb.2022/0972 from Naqb El Farafra as well as specimens B1432, B1433, B1531 and MB.Pb.2022/1049 from Abu Tartur.

Affinity: similar to *Eucalyptolaurus* and "*Myrtophyllum*" [117] in terms of gross morphology but differing by the absence of intersecondaries.

Description: leaves simple; petiolar attachment marginal; lamina notophyllous, L:W ratio >5; lamina ovate, symmetrical, unlobed and untoothed, coriaceous; apex acute, base acute, convex; primary venation pinnate, agrophic veins absent, secondary venation simple brochidodromous, interior secondaries absent, angle of secondaries uniform, less than one intersecondary per intercostal area, higher order of venation unknown.

**Morphotype-09**

Material: specimens B0115 (Figs 8 and 9I) and MB.Pb.2022/0930 from Naqb El Farafra.

Affinity: unknown.

Description: leaves simple; petiolar attachment unknown; lamina mesophyllous, L:W ratio ca 2; lamina elliptical, symmetrical, unlobed and untoothed, chartaceous; apex and base acute; primary venation pinnate, agrophic veins absent, secondary venation simple brochidodromous, interior secondaries absent, angle of secondaries uniform, less than one intersecondary per intercostal area, higher order of venation unknown.

**Morphotype-10** (*Liriophyllum farafraense* Lejal-Nicol et Dominik).

**Fig 10:**
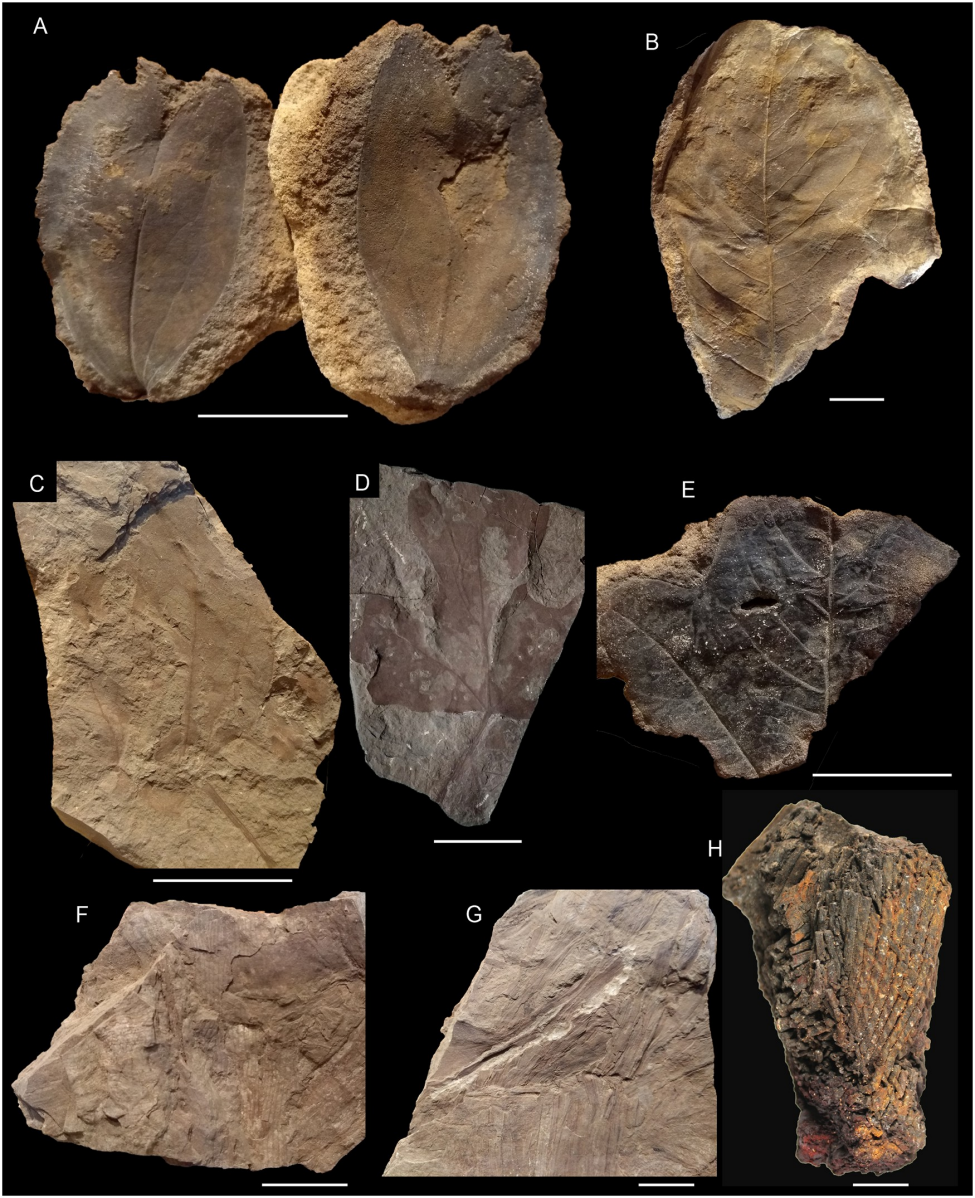
Plant macro-remains from the Cenomanian of Egypt; The scale bar is equal to 2 cm. (A) Morphotype-10 (Naqb El Farafra, B0116), (B) Morphotype-11 (Naqb El Farafra, B0070), (C) Morphotype-12 (Gebel El-Tibnia, MB.Pb.2022/1044), (D) Morphotype-13 (Gebel El-Tibnia, B0006), (E) Morphotype-14 (Gebel El-Tibnia, B0110), (F-G) Morphotype-17 (Gebel El-Tibnia, B0015), (H) Morphotype-18 (Gebel El-Tibnia, MB.Pb.2022/1014), (I) *Paradoxopteris stromeri* Hirmer, 1927 (Gebel El Minshera, SM.B. 22092) (from [33]).

Material: specimen B0116 (Figs 8 and 10A; *L*. *farafraense*) from Naqb El Farafra.

Affinity: unknown, this morphotype represents the holotype of *L*. *farafraense* Lejal-Nicol et Dominik (B116).

Description: leaves simple; petiolar attachment marginal; lamina microphyllous, L:W ratio ca 2; lamina elliptical, symmetrical, unlobed and untoothed, chartaceous; apex reflex, emarginate, base acute, convex; primary venation pinnate, agrophic veins absent, secondary venation simple brochidodromous, interior secondaries absent, one pair of acute basal secondaries, higher order of venation unknown.

**Morphotype-11**

Material: specimens B0070 (Figs 8 and 10B), B0115, MB.Pb.2022/0909, and MB.Pb.2022/0910 from Naqb El Farafra (*Magnoliaephyllum isbergiana* (Heer) Lejal-Nicol et Dominik).

Affinity: the festooned brochidodromous venation may indicate affinities to magnoliids.

Description: leaves simple; petiolar attachment unknown; lamina mesophyllous, L:W ratio unknown; lamina shape unknown, unlobed and untoothed, coriaceous; apex obtuse, base unknown; primary venation pinnate, agrophic veins apparently absent, secondary venation festooned brochidodromous, interior secondaries absent, angle of secondaries apparently uniform, less than one intersecondary per intercostal area, tertiary venation opposite percurrent, perpendicular to the mid-vein, epimedial tertiaries well visible, opposite percurrent, admedial course acute to the midvein and exmadial course parallel to the intercostal tertiaries, higher order of venation unknown.

**Morphotype-12**

Material: specimen B0117 from Naqb El Farafra, as well as specimens MB.Pb.2022/1034 and MB.Pb.2022/1044 from Gebel El-Tibnia (Figs 8 and 10C), and only specimen MB.Pb.2022/1050 from Abu Tartur.

Affinity: similar leaves were referred to *Cinnamophyllum* from the Sabaya Formation (Schrank, 1990). Possible lauraceous affinities?

Description: leaves simple; petiolar attachment unknown; lamina micro- to notophyllous, L:W ratio ca 2; lamina ovate, unlobed and untoothed, chartaceous; apex obtuse, rounded, base obtuse; primary venation acrodromous, agrophic veins simple, major secondary venation simple brochidodromous, interior secondaries absent, minor secondaries simple brochidodromous, angle of secondaries unknown, less than one intersecondary per intercostal area, higher order of venation unknown.

**Morphotype-13**

Material: specimens B0006 and B0022 (Figs 8 and 10D) from Gebel El-Tibnia (both *Vitiphyllum* aff. *multifidum* Fontaine, *Cissites* cf. *insignis* Heer in [70]).

Affinity: similar specimen from Saudi Arabia [118], some similarities with *Pabiana*.

Description: leaves simple; petiolar attachment marginal; lamina mesophyllous, L:W ratio ca 1; lamina ovate, palmately lobed and apparently untoothed, chartaceous; apex unknown, base obtuse, cordate; primary venation basal actinodromous, agrophic veins simple, major secondary venation unknown, interior secondaries apparently absent, minor secondary venation unknown, higher order of venation unknown.

**Morphotype-14**

Material: specimens B0110 (Figs 8 and 10E) from Naqb El Farafra.

Affinity: older “lauroid” leaves, *Araliaephyllum* from the Sabaya Formation [119], *Sassafras brachylobum* Mädler from Jordan [120].

Description: leaves simple; petiolar attachment unknown; lamina at least microphyllous, L:W ratio unknown; lamina shape unknown, apparently palmately lobed and untoothed, chartaceous; apex and base unknown; primary venation actinodromous, agrophic veins simple, major secondary venation unknown, interior secondaries absent, minor secondary venation unknown, higher order of venation unknown.

**Morphotype-15**

Material: specimen B1553 from Naqb El Farafra and specimen B0023 (Fig 8) from Gebel El-Tibnia (*Nelumbites schweinfurthi*).

Affinity: Peltate leaves with numerous primaries are typical of Nelumbonaceae.

Description: leaves simple; petiolar attachment peltate (probably central); lamina probably at least mesophyllous, L:W ratio unknown; lamina ovate, probably unlobed and untoothed, chartaceous; apex and base unknown; primary venation basal actinodromous with at least 10 primaries, agrophic veins absent, major secondary venation probably festooned brochidodromous, interior secondaries apparently absent, tertiaries opposite percurrent, convex, higher order of venation unknown.

**Morphotype-16** (*Nelumbites giganteum* Lejal-Nicol et Dominik).

Material: specimen B0001 (Figs 8 and 10B) from Naqb El Farafra.

Affinity: This morphotype corresponds to the holotype of *N*. *giganteum* Lejal-Nicol et Dominik which is probably not a Nelumbonaceae.

Description: leaves simple; petiolar attachment centrally peltate; lamina at least mesophyllous, L:W ratio ca 1.5; lamina elliptic, unlobed and untoothed, coriaceous (venation in strong relief); apex and base obtuse and rounded; primary venation basal actinodromous with at less than 10 primaries, agrophic veins simple, major secondary venation festooned brochidodromousm, interior secondaries apparently absent, minor secondary venation festooned brochidodromousm, tertiaries opposite percurrent, convex, higher order of venation unknown.

Monocots

**Morphotype-17**

Material: B0015 (Figs 8 and 10F) from Gebel El-Tibnia (cf. *Typhaephyllum* sp.).

Affinity: this specimen was interpreted as a monocot.

Description: Leaves simple parallel-veined without further diagnostic character.

**Morphotype-18**

Material: only specimen MB.Pb.2022/1014 from Gebel El Tibnia (Figs 8 and 10G).

Affinity: some similarities with *Plumafolium* from the Turonian of the Negev [121].

Description: petiolate leaf bases with fan-like parallel venation.

Ferns:

**Morphotype-19** (*Weichselia reticulata* (Stokes et Webb) Fontaine).

Material: one specimen from Gebel El-Tibnia.

The foliage of the fern *Weichselia reticulata* is widely distributed in the late Early and early Late Cretaceous of Northern Africa and the Middle East (cf. [33] and citations therein). Many authors recognized this taxon as a typical constituent of coastal vegetation (e.g., [34,60,122–126]) or even as an element of mangrove-like vegetation (e.g., [16,34,124,126,127]) but it is also known from a number of inland habitats (e.g., [128–130]).

**Morphotype-20** (Fig 10H; *Paradoxopteris stromeri* Hirmer 1927)

Permineralized remains of *Paradoxopteris stromeri*, representing the rachis/petiole/stem of the fern *Weichselia reticulata* are known from a number of Cenomanian localities in Egypt (e.g., [12,16,33,61,131,132]). Most of these records, including the type-material, come from deposits of the Bahariya Formation (e.g., [16,131,132]), but a few are also known from the Galala Formation in Sinai (e.g., [33,132]).

### 4.2. Floristic compositions and comparisons; megaflora results

The assemblages of the Bahariya and Maghrabi formations are very similar and dominated by rather stereotyped morphotypes (simple, entire-margined, pinnately veined leaves) (Figs 9 and 10). Nevertheless, the disparity and diversity of higher-order venation indicate a relatively diverse flora. Their physiognomy is in contrast to northern mid-latitude assemblages of a similar age such as the Bohemian or Potomac floras which display a much broader spectrum of leaf form (in terms of lobation, teeth, compound leaves, [119]). Furthermore, it seems that no taxa in common with these assemblages are found, due to the small sample size and lack of preservation of the higher-order venation, it is hard to demonstrate this based on the current collection.

The Naqb El Farafra and Abu Tartur profiles correspond to fluvial ferruginous sandstones and are dominated by morphotypes 7 and 8 while Gebel El-Tibnia resembles claystones and is dominated by morphotypes 12 and 13. Furthermore, monocots and aquatics represent one-third of the specimens of the latter assemblage. This associated with the parautochtony supported by the low level of fragmentation, suggests that these localities represent two different floral assemblages, Naqb El Farafra and Abu Tartur assemblages likely representing riparian vegetation and Gebel El-Tibnia representing more paludal vegetation characterized by the frequency of aquatics. The *Weichselia* specimens are embedded in grey siltstone facies differing from both the Naqb El Farafra and Gebel El-Tibnia. This species was also reported from a mangrove palaeosoil in the Bahariya area [32] and from a large number of other localities all over North Africa and the Middle East (cf. [33,133]; and citations therein).

Despite the poor preservation of the Cenomanian wood from the Bahariya Formation, it was clear that there was already a flora rich in deciduous trees [12]. However, [14] stated that plant remains in the Cenomanian Bahariya Formation were deposited under lagoonal and intertidal settings and that some remains would advocate for a long transport from inland forests although others would indicate close vicinity, based on taphonomic considerations. They also inferred that the existence of freshwater taxa like *Nelumbites* and floating aquatic ferns in such salt-water-influenced conditions denote that freshwater ponds were present in paralic environments. Furthermore, they mentioned that the form genera recorded from Bahariya are similar to those from North American Cenomanian megafloras (e.g., Dakota Group Flora) and that leaf physiognomy shows that the area was comparatively dry and warm at that time. However, these interpretations should be treated as preliminary as they were presented only in an abstract [14]. By contrast, we interpret the climate as rather humid based on the relatively large leaf size and we did not find affinities with northern mid-latitude flora based on the detailed re-description of the material.

Outside Egypt, the flora of the Umm Badda Member of the Omdurman Formation [119] is quite similar to the Bahariya assemblage, being dominated by angiosperms of mesophyllous size [134], but further taxonomic revision and collection of additional material, is necessary to better understand their affinities. In contrast to the assemblages of Egypt and Sudan, the Albian-Cenomanian assemblages from Lebanon and Morocco are dominated by ferns and gymnosperms [135,136]. Angiosperms of these assemblages are of nanno- to microphyllous size. Nevertheless, younger assemblages (Turonian-Santonian?) from Jordan include at least one morphotype with affinities to the Egyptian assemblages, i.e., *Sassafras brachylobus* Mädler [120]. It is therefore important to consider that the present state of the taxonomy of the Egyptian macroflora assemblages at most is tentative.

#### 4.2.1. Climate

The dominance of simple, entire-margined leaves suggests a tropical warm climate, which is in agreement with a palaeoposition of Egypt between ~10 and 25°N (Fig 2), and in terms of leaf size, the dominance of mesophyllous morphotypes suggests a rather humid climate [137]. A preliminary estimate of the precipitation, based on only 14 morphotypes (assuming that all of these morphotypes represent arborescent dicots) having an average area of 37 cm^2^ gave ca 2000 mm precipitation per year (1300–2800 mm) based on [116] regression analysis and about 1600–1700 mm based on both regressions used in [138]. However, it has to be kept in mind that smaller leaves have a higher potential to enter the fossil record than larger leaves (e.g., [139]), thus such an estimate of precipitation based on leaf size alone might be too low.

A review of previous palynological studies on Egyptian samples has revealed that many authors assumed a warm tropical to subtropical, arid to semi-arid climate for the Bahariya Formation and its equivalents (e.g., [18,19,24–27]). In addition, dry phases occurred in NE Africa during the Cenomanian and minor humid phases were also recorded during the Albian-Cenomanian, as evidenced by the distribution of xerophytic versus hygrophilous palynoflora [82]. Similarly, [140] stated that the climate of northern Gondwana is palynologically interpreted as tropical but semi-arid, low humidity is reasoned from the richness of *Ephedra* and the low diversity of pteridophytic spores.

From our own investigation, this assumption needs to be revised, as it is generally not in agreement with the well-known high biomass ecosystem, especially what is already known from Gebel El Dist Member (Bahariya Formation), as discussed earlier. This contradiction of palynological data with current results gained from leaves could be attributed not only to the lack of detailed quantitative data but also to sampling error, low sample size or even the interpretation of the climate in a rather general context by allocating Egypt to the Albian-Cenomanian Phytogeographic Province of [29] and Gebel El Dist Member is a case study of the Bahariya Formation. However, few attempts assumed the presence of relatively humid interphases within this arid climate (e.g., [28,141]) which relatively increase up-section within the Bahariya Formation [142]. Furthermore, the occurrence of fossil tooth plates of the lungfish *Ceratodus* in the Bahariya Formation [39] could be seen as an indication of seasonality, as this fish genus can inhabit arid zones due to its capacity for activation during dry periods [143]. The understanding of depositional environments of the Bahariya Formation, based on microfacies analyses and sequence stratigraphy [144] reveals that a gradual transition between different facies associations reflects a climate change from drier to wetter conditions.

### 4.3. Palynological results (Bahariya Formation)

Our palynological investigations have shown that most of the investigated samples are palyniferous and yielded all in all a rich, diverse, and stratigraphically relevant assemblage of palynomorphs to present an overview of the palynoflora (Table 2), both marine (mainly dinoflagellate cysts) and non-marine. A representative group of spores and pollen taxa are illustrated (Figs 4–7). Within the current investigation, cholorococcalean algae, spores, and pollen were identified. Algae are assigned to two families, in addition to *Schizosporis reticulatus*, spores to 8 families, and pollen grains to 13 families. Sporomorph taxa are categorized into the following groups: ferns, gymnosperms (gnetaleaen and non- gnetaleaen), and angiosperms (Table 3). For each category, the fossil genera are arranged in alphabetic order (Table 2). Botanical affinity and geographic occurrences of extant analogues, ecology, and palaeofloristic implications are outlined for each taxon, when possible (Table 3). The total assemblage (excluding dinocysts and other marine elements) consists of 49 genera belonging to fern spores (11 genera), gymnosperm (15 genera), and angiosperm pollen including *Afropollis* (20 genera), and algal remains (3 genera).

Ferns are mainly represented by Filicopsida; Cyatheaceae (*Cyathidites*), Lygodiaceae (*Triplanosporites*), Matoniaceae (*Cibotiumspora jurienensis*, *Concavisporites*, *Trilobosporites*, *Matonisporites*), Schizaeaceae, Anemiaceae (*Cicatricosisporites*), and Incertae Sedis (*Leptolepidites*), aquatic ferns include Marsileaceae (*Crybelosporites pannuceus*, *Gabonisporis vigourouxii*) and Salviniaceae (*Ariadnaesporites*). Non-gnetalean gymnosperms comprised Erdtmanithecaceae (*Eucommiidites*), Cycadales, Ginkgoales, and Bennettitales (*Cycadopites*, *Exesipollenites*, *Monosulcites*) and conifers. Among the conifers, representatives of Cheirolepidiaceae (*Classopollis*) are by far the most abundant, followed by Araucariaceae (including *Araucariacites* and *Balmeiopsis*). Gnetalean gymnosperms are represented by Elaterate Complex (*Elaterocolpites*, *Elateroplicites*, *Elaterosporites*, *Senegalosporites*, *Sofrepites*) and Ephedroides (*Ephedripites*, *Equisetosporites*, *Steevesipollenites*). Angiosperms are relatively rare but show a progressive increase in species richness comprising eudicots, monocots, and dicots (Table 3). Moreover, *Afropollis*, which might even represent an unknown group of seed plants and not necessarily an angiosperm ([1,145,146]) is the most dominant taxon. The palynological yield from these productive samples can be summarized as follows:

#### 4.3.1. GPJ-1 Well

Five productive cutting samples are investigated from this well, their yield is as follows: Spores (ferns): *Cibotiumspora jurienensis*, *Concavisporites* spp., *Cicatricosisporites minutaestriata*, *Cicatricosisporites* sp., *Cyathidites australis*, *Cyathidites minor*, *Triplanosporites* sp., aquatic ferns include *Crybelosporites pannuceus*. Gymnosperms: *Araucariacites australis*, *Cycadopites* sp., *Ephedripites* spp., *Eucommiidites minor*, *Eucommiidites troedssonii*, *Steevesipollenites* cf. *binodosus* and elaterates (*Elaterocolpites castelainii*, *Elateroplicites africaensis*, *Elaterosporites klaszii*). Angiosperms: *Cretacaeisporites densimurus*, *Retimonocolpites* sp., *Retitricolpites* sp., and *Tricolpites* sp., as well as *Afropollis jardinus* and *Afropollis kahramanensis*.

Marine palynomorphs include dinocysts such as *Florentinia mantelli*, *Clesitosphaeridium* sp., *Coronifera albertii*, *Cyclonephelium vannophorum*, and *Xiphophoridium alatum*, in addition to acritarchs and microforaminiferal linings.

#### 4.3.2. TSW-21 well

This well yielded thirteen productive (three core + 10 cuttings) samples. They contain the chlorococcalean algae *Botryococcus* and *Pediastrum*. Sporomorph taxa include: Spores (ferns): *Cibotiumspora jurienensis*, *Concavisporites* spp., *Cicatricosisporites minutaestriata*, *Cicatricosisporites* sp., *Cyathidites australis*, *Cyathidites minor*, *Matonisporites* sp., *Triplanosporites* sp., aquatic ferns include *Ariadnaesporites* sp., *Crybelosporites pannuceus* and *Gabonisporis vigourouxii*. Gymnosperms comprised *Araucariacites australis*, *Balmeiopsis limbatus*, *Classopollis brasiliensis*, *Classopollis* spp. (including *Corollina* and *Circulina*), *Cycadopites carpentieri*, *Cycadopites* sp., *Ephedripites jansonii*, *Ephedripites* spp., *Equisetosporites ambiguus*, *Eucommiidites minor*, *Eucommiidites troedssonii*, *Exesipollenites* sp., *Monosculcites* sp., *Steevesipollenites binodosus*, *Steevesipollenites* cf. *binodosus* and elaterates (*Elateroplicites africaensis*, *Elaterosporites klaszii*). Angiosperms are represented by *Albertipollenites rosalindiae*, *Cretacaeisporites densimurus*, *Cretacaeisporites polygonalis*, *Cretacaeisporites scabratus*, *Dichastopollenites dunveganensis*, *Foveotricolpites giganteus*, *Monocolpopollenites* sp., *Nyssapollenites* sp., *Pennipollis peroreticulatus*, *Retimonocolpites variplicatus*, *Retitricolpites* sp., *Rousea* sp., *Stellatopollis* sp., *Syncolpites* sp., *Tricolpites* sp., and *Tricolporopollenites* sp., as well as *Afropollis jardinus* and *Afropollis kahramanensis*.

Marine palynomorphs include dinocysts such as *Cribroperidinium edwardsii*, *Florentinia mantelli*, *Clesitosphaeridium* sp., *Circulodinium distinctum*, *Coronifera albertii*, *Coronifera tubulosa*, *Oligosphaeridium complex*, and *Spiniferites* sp., in addition to acritarchs and microforaminiferal linings.

#### 4.3.3. GPT-3 well

Four productive cutting samples are collected from the Bahariya Formation of this well. The recorded palynomorphs are mostly terrestrial, in addition to a minor marine input of dinocysts and microforaminiferal linings, as well as a single scolecodont specimen. Chlorococcalean algae comprise *Botryococcus* and *Pediastrum*. Sporomorphs taxa include: Spores (ferns): *Concavisporites* spp., *Cyathidites australis*, *Cyathidites minor*, *Triplanosporites* sp., aquatic ferns include *Crybelosporites pannuceus*, and a single record of the megaspore *Ariadnaesporites* sp. Gymnosperms comprised *Araucariacites australis*, *Classopollis brasiliensis*, *Classopollis* spp. (including *Corollina* and *Circulina*), *Ephedripites jansonii*, *Ephedripites* spp., *Eucommiidites minor*, *Eucommiidites troedssonii*, *Monosculcites* sp., *Steevesipollenites* cf. *binodosus* and elaterates (*Elaterosporites klaszii*). Angiosperms are represented by *Cretacaeisporites aegyptiaca*, *Cretacaeisporites densimurus*, *Integritetradites porosus*, *Nyssapollenites* sp., *Retimonocolpites* sp. and *Retitricolites* sp., as well as *Afropollis jardinus* and *Afropollis kahramanensis*.

Marine palynomorphs include dinocysts such as *Circulodinium distinctum*, *Cribroperidinium* spp., *Clesitosphaeridium* spp., *Dinopterygium cladoides*, *Florentinia* spp., and *Subtilisphaera* spp., as well as microforaminiferal linings (see [73]).

#### 4.3.4. GPTSW-7 well

Thirty-four cutting samples representing the Bahariya Formation, retrieved from the well GPTSW-7 are palyniferous. The palynofloras are dominated by terrestrially-derived palynomorphs. They are represented by chlorococcalean algae comprising *Botryococcus*, *Pediastrum*, and *Schizosporis reticulatus*. Sporomorph taxa include: Spores (ferns): *Cibotiumspora jurienensis*, *Concavisporites* spp., *Cicatricosisporites minutaestriata*, *Cicatricosisporites* spp., *Cyathidites australis*, *Cyathidites minor*, *Dictyophyllidites harrisii*, *Leptolepidites* spp., *Triplanosporites* sp., *Trilobosporites laevigatus*, aquatic ferns include *Ariadnaesporites* sp., *Crybelosporites pannuceus*, and *Gabonisporis vigourouxii*. Gymnosperms comprised *Araucariacites australis*, *Balmeiopsis limbatus*, *Classopollis brasiliensis*, *Classopollis* spp. (including *Corollina* and *Circulina*), *Cycadopites carpentieri*, *Cycadopites nitidus*, *Cycadopites ovatus*, *Ephedripites* spp., *Monosculcites mimimus*, *Monosculcites* sp., *Steevesipollenites grambasti*, *Steevesipollenites sinusous*, *Steevesipollenites* cf. *binodosus* and elaterates (*Elaterocolpites castelainii*, *Elateroplicites africaensis*, *Elaterosporites klaszii*, *Senegalosporites petrobrasi*, *Sofrepites legouxae*). Angiosperms are represented by *Albertipollenites rosalindiae*, *Cretacaeisporites aegyptiaca*, *Cretacaeisporites densimurus*, *Dichastopollenites ghazalatensis*, *Foveomorphonocolpites rashadi*, *Foveotricolpites giganteus*, *Integritetradites porosus*, *Nyssapollenites* sp., *Proteacidites* cf. *africaensis*, *Retimonocolpites variplicatus*, *Retitricolporites pristinus*, *Tetracolpites* sp., and *Tricolpites* sp., as well as *Afropollis jardinus* and *Afropollis kahramanensis*.

Marine palynomorphs, however, out of our focus, are in lesser amounts and dominated by dinoflagellates, smooth- and thin-walled acritarchs as well as microforaminiferal linings, all of which are completely listed in [62,72].

### 4.4. Climatic and vegetational development

**Fig 11:**
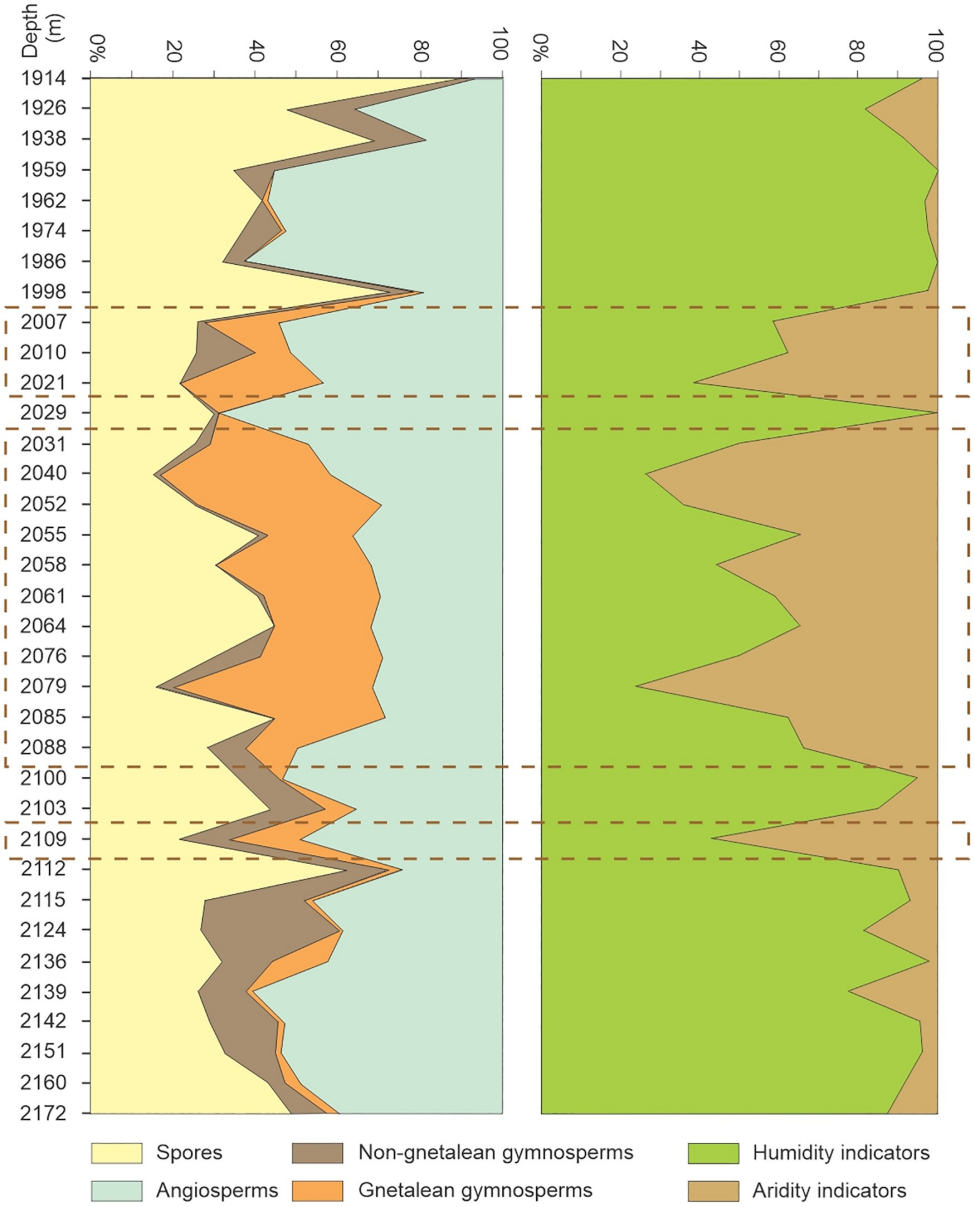
Climate data obtained from the GPTSW-7 well, shows a more arid phase, coinciding with the abundance of Gnetales.

Based on palynological samples from the well GPTSW-7, it is possible to provide data about the climatic and vegetational development during the deposition of the Bahariya Formation (Table 2, Fig 11). The other boreholes did not yield enough palyniferous samples to allow for a meaningful interpretation through time. However, their close spatial and temporal situation makes the GPTSW-7 representative of a reliable regional scenario.

Aridity indicators are very rare spanning the interval 2,171 m to 2,100 m with one exception recorded at a depth of 2,109 m (Table 2, Fig 11). At depth 2,088 m aridity indicators increase and stay high, with smaller oscillations, up to 2,007 m.

This drier phase, coinciding with a phase of markedly higher abundance of gnetalean gymnosperms as compared to the rest of the section, is interrupted at 2,009 m, by a sample containing no gnetalean pollen, as well as other aridity indicators. After 2,009 m aridity indicators stay generally low, with minor fluctuations in abundance. This indicates that during the deposition of the Bahariya Formation in the area of GPTSW-7 a more humid phase was interrupted by a longer phase with drier conditions, which was punctuated by short phases again with a more humid climate.

During drier phases, humidity indicators probably shifted their ranges more to the south, as recognized by the higher spores frequency in samples from southern Egypt (47%: [147]; up to 45%: [148]; present-abundant: [26]). However, other results from southern Egypt, such as [27] did not trace such a trend as no counting was done. In their study on the equivalent Maghrabi Formation of the Dakhla Oasis [65], postulated the existence of possibly seasonal dryness which forestalled the high diversity of the palynoflora.

Recently [21], studied subsurface samples from the Bahariya Formation encountered in the GPC concession, Abu Sennan area (Fig 1) which is close to our studied boreholes. Although this work is of biostratigraphic nature, they emphasized the occurrence of a clear cyclicity of gnetalean taxa (see Supplementary data). This led them to assume that the abundance of these gnetalean palynoflora pinpoints a drier phase with more prolonged dry spells. This interpretation is corroborated by our results.

In this respect, *Afropollis jardinus* in our material displayed a proliferation peak (Table 2). Such a peak has been repeatedly recognized in Egypt (e.g., 11–64%: [149]; 31–60%: [150]; 24–62%: [28]). This *Afropollis jardinus* peak has been interpreted by [141] to be controlled by local humid conditions. Outside Egypt, such a peak was also recorded from Senegal [151].

Unlike studies carried out in temperate regions, the effects of desert weathering and associated oxidative destruction on outcrop sections in Egypt have serious implications for palynological investigations. It is evident that most of the surface exposures of Mesozoic deposits in Egypt are largely devoid of palynomorphs (e.g., [24]). It was, therefore, necessary to select alternative subsurface sections for investigations and this makes it difficult to establish an accurate temporal as well as a spatial correlation between well and surface data. In addition, the base of the Bahariya Formation in the surface exposures even in its type section (Gebel El Dist profile) is not exposed. However, our data offer a tentative correlation based on the distribution of climatic-diagnostic palynomorphs together with their relative megafloral elements gained from both surface and subsurface data, as well as data from [17] and [23]. At least climatically, the macroflora from the Gebel El Dist profile, indicating a high biomass ecosystem that sustained the giant herbivore and carnivore dinosaurs, correlates very well with the lower part of the well GPTSW-7, mainly with *Cretacaeisporites densimurus* subzone of [62] and its counterparts.

## 5. Discussion

### 5.1. Depositional environment

Differences in depositional settings can have a large significance on parent vegetation and resultant sporomorph assemblages and/or preferential preservation of certain sporomorph taxa due to taphonomic processes. Moreover, different depositional environments frequently display substantial variation concerning the geographical basins that supply deposits [99]. The Bahariya Formation was deposited in a shallow marine and fluvio-deltaic setting [152], or shallow inner shelf (0–50 m water depth) as a result of a regressive phase [140]. Previous palynological evidence (e.g., [19]) assumed its deposition in deltaic to shallow marine nearshore environment. However, preceding palynofacies results [62] indicate that the Bahariya Formation was deposited in a shallow marine environment with a high terrestrial or freshwater influx.

The recorded palynofloras are dominated by terrestrially derived spores, gymnosperm, and angiosperm pollen (Figs 4–6), in addition to the paucity of marine palynomorphs, which indicate deposition in a continental environment, and proximity to the shoreline. Miospores are represented mostly by fern spores. The gymnosperm pollen is dominated by conifer vegetation developed on the comparatively dry hinterland, accompanied by the occurrence of ephedroides, elaterates, and *Classopollis*, largely interpreted as xerophytes [65]. Evidence from macroflora indicates a nearly worldwide distribution of araucarian trees, however, they often grew in closer forests not far from the coast [153,154], therefore the high frequency of *Araucariacites* may be an indication of coastal ecology [80].

The persistent occurrences of the aquatic fern spores *Ariadnaesporites*, *Crybelosporites*, and *Gabonisporis* growing adjacent to or in freshwater bodies [155], give support to the occurrence of swamps or water-logged environments. Macrofloral elements probably represent water plants such as *Nelumbites* that were also recorded from the investigated sections in the Bahariya Oasis as well as south to the northeast of Wadi Halfa [59]. Similar palynological data was also recognized from the southern equivalent Maghrabi Formation of the Dakhla Oasis by [65] as well as from the El-Heiz Formation in the Farafra Oasis [156], who suggested the occurrence of freshwater lakes and ponds during the deposition. Later, [26] proposed that the Maghrabi Formation was deposited in a marginal marine setting influenced by high fresh water and land-derived material influx.

Furthermore [141], linked the existence of elaterates and *Afropollis* to shallow marine settings. The large trees of the Cheirolepidiaceae (*Classopollis*) are typical of dry flood-plain settings [99]. However, *Frenelopsis* (Cheirolepidiaceae) is a typical constituent of salt marshes in the Late Cretaceous of Europe [157]. To the best of our knowledge, Cheirolepidiaceae had a broad ecological amplitude in the Early Cretaceous (e.g., Las Hoyas; [158]) but after the Albian, their records seem to be restricted to salt marshes (e.g., western France, Peruc Formaion; [159]).

In addition to the obviously aquatic taxa (e.g., *Nelumbites*), the presence of dispersed dicot leaves with possible lauroid affinities (i.e., morphotypes 13 and 14), suggests the presence of woody plants that could correspond to swamp forests.

Dinoflagellate cysts, despite their preservation in smaller amounts, are dominated by the genus *Cyclonephelium* which indicates a coastal to nearshore environment [160,161].

We interpret the combination of the recorded palynoflora, macroflora as well as charcoal (cf. [8]) as evidence of deposition in fluvial, coastal to nearshore environments affected by warm and arid climates. Furthermore, palynofacies and geochemistry data indicated that the Bahariya Formation was deposited in a proximal suboxic to oxic shallow marine to fluvio-deltaic environment, where high terrestrial/fresh-water input was mixed with marine organic matter [62,73].

### 5.2. Vegetation and floristic composition and palaeoclimate

#### 5.2.1. Megaflora composition

The previous plant remains recorded from the Bahariya Formation include pteridophyte leaves and rhizomes/rhachis (*Weichselia reticulata*, *Paradoxopteris stromeri*), gymnosperms (Araucariaceae, Cycadaceae, Ginkgoaceae, Taxodiaceae), dicot leaves (*Aquatifolia*, *Araliaephyllum*, *Avicennia*, *Celtis*, *Cornaephyllum*, *Dipterocarpophyllum*, *Ficophyllum*, *Ficus*, *Laurophyllum*, *Magnoliaephyllum*, *Nelumbites*, *Nelumbo*, *Nymphaea*, *Populus*, *Rogersia*, *Salix* and *Vitiphyllum*) and leaves and fruits of monocots (Arecaceae and Poaceae), as well as other fossil plants known to be mangrove or mangrove associates (e.g., [11,12,15,16,57–61]).

Nevertheless, except for *Weichselia*, *Paradoxopteris*, and to a lesser extent *Nelumbites*, the generic determination of the material is seen as highly speculative. The megaflora is dominated by angiosperms and displays a strong similarity between Naqb El Farafra and Abu Tartur. Both localities are consisting of fluvial deposits. These assemblages are characterized by simple, entire margined, pinnate leaves, usually without fragmentation. The parautochthony associated with the fluvial character of the sediments suggests that these assemblages represent riparian vegetation. The morphotype 16, occurring in Naqb El Farafra could represent a reophytic aquatic plant.

The Gebel El-Tibnia locality differs from Naqb El Farafra and Abu Tartur in terms of floristic composition and sedimentology. In terms of floristic composition, this assemblage is dominated by aquatics such as *Nelumbites schweinfurthii* (morphotype 15) and monocots (morphotypes 17 and 18) which may have been helophytic (i.e., emergent water plants like *Pontederia*), and interpreted to be a swamp based on floral assemblages and previous sedimentology data.

Despite the low diversity and sparse palynomorph data of [17,23], it is evident that the integration of their data with ours, at least climatically, offers a tentative correlation between palynomorph and megafloral elements in both surface and subsurface data (Fig 11). Following such an approach, the overall physiognomy of the megaflora, dominated by mesophyllous and entire margined leaves, suggests a rather humid tropical climate that would fit with the humid phase of the lower part of the GPTSW-7 section, or which could be caused by edaphic factors, like high groundwater tales in swamps and riparian habitats.

#### 5.2.2. Palynofloristic composition

The recovered sporomorphs were grouped into four plant categories to help in the interpretation of the floristic composition: spores, gnetalean gymnosperm pollen, non-gnetalean gymnosperm pollen, and angiosperm pollen. Gymnosperms were divided into non-gnetalean and gnetalean since the latter are significant climate indicators and represent an important component of many Cretaceous floras [81]. In our samples, the non-gnetalean gymnosperm pollen embrace conifers, and monosulcate pollen grains such as those of Cycadales and Bennettitales. The floristic configuration of the Bahariya Formation was determined from palynomorph abundance (Table 2). However, additional taxa that are floristically diagnostic, and absent in our material will also be discussed.

The degree of aridity *versus* humidity was established by using indicator species that have been recognized to favour a given environment based on known climatic preferences of their extant relations. Indicators of aridity include *Classopollis*, ephedroid pollen, and elater-bearing species, while the indicators of humidity include mainly fern spores. This assignment treats all spores collectively as humidity indicators; however, some species might have belonged to arid-adapted ferns (e.g., [81]), thus we additionally applied the SEG technique to produce an improved scenario for the climatic inferences.

In our assemblages, chlorococcalean algae comprise Botryococcaceae (*Botryococcus*), Hydrodictyaceae (*Pediastrum*), and *Schizosporis reticulatus*. Sporomorph assemblages are dominated by ferns, conifers, and *Afropollis* with minor changes in composition through the studied boreholes.

To the best of our knowledge, previous palynofloral elements described from surface sections in the Cenomanian were exclusively available from the Gebel Dist (Bahariya Oasis) and yielded early Cenomanian palynoflora [17], however, a late Cenomanian (or Turonian?) age may be assumed for this likely angiosperm-dominated palynoflora that contains the most advanced angiosperms, triporates and perhaps also periporate pollen related to *Cretacaeisporites* (Pl. 1-Fig 6 in [17]) as well as normapolles taxa [23]. *Deltoidospora* and other more or less smooth triletes, *Ephedripites*, and *Tricolpites* also take part in this palynoflora. It is difficult to correlate this profile with any other surface or subsurface sections, as no stratigraphic data were available ([17,23]). Furthermore, [24] identified palynomorph assemblages from the Cenomanian Galala Formation at Gebel El Minshera, north Sinai, despite rare, a pteridophytic spore-dominated assemblage with a single *Afropollis jardinus* record was topped by the abundant and monospecific occurrence of the brackish to coastal dinocyst taxon *Subtilisphaera*.

Our interpretations of palaeoclimate are consistent with previous investigations of Cenomanian pollen records from Egypt ([18–22,28,62,67,72,140–142,149,150,162–174]). Some studies have reported palynomorph taxa that are not present in our samples [23,26,28,170,172,173,175], as highlighted in Table 3. Although a lot of data has so far been gathered for individual localities focusing on different aspects of macro-palaeobotany and palynology, there is still a need to present a more detailed, holistic scenario on the vegetation and climate development using different lines of evidence (cf. [176]) to better understand the Cenomanian ecosystem which locally hosted a rich fauna.

An early Cenomanian (to late Albian?) palynoflora described by [175], from the Ammonite 1 well lacks typical *Afropollis* and elaterate pollen; similarly, the assemblages described by [177,178] also lack elater-bearing taxa, both from the subsurface of the north Western Desert. Their absence in Egyptian strata was first documented by [67] and this may be related to palaeoecological issues, different flora, or insufficient sampling of investigated strata. Later, [141] suggested that *Afropollis* and elaterate producers are largely inhabitants of palaeotropical humid coastal plains. In addition, [25] described palynofloral assemblages of the Raha Formation from the subsurface Gulf of Suez. Their assemblage totally lacks elaterates, however, low diversity of spores (mainly ferns), gymnosperm pollen (*Araucariacites*, *Classopollis*, *Cycadopites*, *Ephedripites*) in addition to angiosperms of *Tricolpites*, and *Retimonocolpites*, as well as *Afropollis* is reported which means that elaterate absence is not an exclusive criterion for the Western Desert and can be spatially repeated.

In southern Egypt, [179] interpreted pollen and spore assemblages from the Bulaq 12 and 15 wells (Kharga Oasis) as Cenomanian which produced highly diverse angiospermous elements, i.e., *Tricolpites*-like pollen. Palynofloral assemblages described from the Um Sidida Formation, Gebel Abraq area, Aswan [27], are dominated by angiosperms and contain fewer spores (mainly ferns) and gymnosperms (*Araucariacites/Inaperturopollenites*, *Cycadopites*, *Taxodiaceaepollenites*), with the advent of Microforaminiferal test linings and acritarchs. Angiosperms are mostly related to *Tricolpites*, *Tricolpopollenites*, and lesser amounts of *Cretacaeisporites polygonalis* and *Trichotomosulcites*. Assemblages described from the subsurface material near Gebel Marawa, Kalabsha by [147] witnessed a dominance of 47% spores (mainly ferns), 35% gymnosperms (mainly 19% inaperturate, 6% *Ephedripites*, 4% *Classopollis*, 3% *Monosulcites*, and 2% *Eucommiidites*) and 18% angiosperms (including *A*. *jardinus*). The age of this assemblage is assigned to the late Albian-early Cenomanian. They (op. cit.), recorded from the same material a younger assemblage (late Cenomanian) that witnessed no marked floral change (21% fern spores, 12% inaperturate, 13% *Ephedripites*, 2% *Classopollis*, 5% *Monosulcites*) as compared to the underlying horizon, except for a higher angiosperm diversity, represented by *Proteacidites* sp., *Triorites* sp. and *Nyssapollenites albertensis*. The most significant palynofloral event is the absence of elaterates, which was also the case for other assemblages from the Maghrabi Formation within the Kharga Oasis [26,65]. Similar results were also gained from southeast Aswan [148], as reported earlier from the north Western Desert assemblages.

The Maghrabi Formation palynoflora of the Dakhla Oasis [65] is characterized by ferns (including Salviniaceae), dominant *Araucariacites* and angiosperms, specifically tricolpates (*Foveotricolpites*), tetracolpates (*Tetracolpites*) and tricolporates (*Tricolporopollenites*, *Nyssapollenites*). It is possible that the material from the Kharga 1 well at 28 and 12 m [68], represents the Maghrabi Formation indicating a post-early Cenomanian age. The occurrence of *Proteacidites* Type A Saad and Ghazaly, 1976; (zone III) would be in accordance with an age not older than late Cenomanian. However, a resemblance of zone III [68] with the Maghrabi palynofloras [65], emphasized their close similarity, and an age older than Santonian-Campanian seems plausible. This fits well with the late Cenomanian age given earlier for zone III [68] and with the middle assemblage zone, dated also as Coniacian, less probably Turonian [69] described from the Bulaq 15 well, south of Kharga Oasis.

It is clear that the palynofloras from the Bahariya Formation in the studied material and previous findings in the north Western Desert and its other counterparts from Egypt are similar and represent part of the Albian-Cenomanian Province of [29] and its equivalents from the coastal basins of West Africa and South America. However, the northern part of this province has been extended as far as Europe, comparison with the assemblage recorded from the Cenomanian of Bohemia [180] also suggests a similar age to the palynofloras of the north Western Desert of Egypt.

#### 5.2.3. Comparison with the plant megafossil record

In comparison to the palynological record, which represents a mix of local and regional vegetation, the macrofossils are a more reliable snapshot of local vegetation (e.g., [176] and citations therein). However, the occurrence of *Cretacaeisporites* in the Gebel El Dist profile and the fact that this genus seems to be restricted within the lower part of the GPTSW-7 borehole, we suggest that this part of the record is more or less synchronous and could be correlated with the macroflora. Interestingly, this part of the column corresponds to the period before the phase of increased aridity. It is also marked by the non-gnetalean gymnosperms which appear to be more common before the arid phase. This lower part of the GPTSW-7 borehole is dominated by *Afropollis* which constitutes nearly half of the palynomorphs, while gymnosperms about one-eighth. Water ferns are also among the palynofloral elements as previously mentioned.

All in all, we infer that the mesophilic macrofossil record could correspond to this wetter phase, however more palynological and macrofloral data from Gebel El Dist are needed to validate our scenario. But unfortunately, [17] lacks detailed counting and comprised an infrequent assemblage of the Chlorophyta alga (*Schizosporis cooksoni*), pteridophytes, *Classopollis*, *Ephedripites*, and angiosperms. Similarly, a palynological investigation carried out on the equivalent Galala Formation from the surface section at Gebel El Minshera [24], yielded a rare, pteridophytic spore-dominated assemblage with a single *A*. *jardinus* record. Here *Paradoxopteris stromeri* was recently reported from a very adjacent stratigraphic layer within the same section [33].

#### 5.2.4. Vegetation analysis

Vegetation categories represented in the Bahariya Formation palynoflora are proposed based on the comparison between botanical affinities of fossil taxa and the most typical plants reported, as well as comparison with previously recorded taxa. Overall, the recorded palynoflora from the Bahariya Formation comprises elements belonging to tropical vegetation, for instance, tropical deciduous forest, open landscapes (dominated by herbaceous plants and low scrubs), and (semi-)arid tropical scrub, in which angiosperms are key elements; besides, more temperate vegetation types may have existed.

Ferns belonging to Anemiaceae, as well as Matoniaceae, are typical for both macro- (*Gleichenia*, *Anemia*, *Weichselia*) and palynofloras (*Appendicisporites*, *Cicatricosisporites*, *Dictyophyllidites*, *Matonisporites*) in the Cretaceous of Western Europe and North America [153]. Moreover, the Anemiaceae are most diverse in recent tropical areas of Australasia. Living taxa flourish in open environments with well-drained soils low in nutrients [88,181].

The group of ephedroids is usually allied with subtropical regions that can display more arid climates [182], however, aridity may have been more predominant in seasonally wet zones during the Cretaceous greenhouse/hothouse climate. On the contrary, a more humid climate for the ephedroid pollen group has been proposed [151].

The elaterate group appeared in the late Albian and flourished for a short-term through the Cenomanian with a diverse advent before becoming virtually instantaneously non-existent in the early Turonian [66]. A noticeable co-existence of elaters with ephedroids at sub-tropics of annual precipitation maxima has been demonstrated [183,184]. As reported by [185], the similarity of ektexine and wall sporoderm structure in these two sets shows a mutual evolutionary basis.

According to [186], a worldwide marginal marine strandline environment of the elaters might also clarify their capacity to spread a long way eastward over the southern borders of the Tethys. This also involves ‘island hopping’ by building a particular casual Cenomanian sub-tropical position of practicably existing continental environs like the northern margin of the now-subducted Indian subcontinent. There may have been other likely stepping-stones of mid-ocean ridge islands occurring in association with the previous spreading midpoint between Australia and India, and other margins of the then-low latitude southern shoreline of Tethys, to Papua New Guinea (PNG). They behave clearly as inconsiderable constituents of the PNG assemblages, dominated by dinocysts associated with a low diversity of other terrestrial sporomorph taxa. Such a palynofloral association added robust evidence of a strandline habitat for the elaterates parent plants.

The palynofloras recovered from the Cenomanian of the north Western Desert (Egypt) are not homogeneous. This reflects a diverse array of depositional environments which doubtless designates a variety of plant communities.

The interpreted vegetation types include 1) freshwater environments as wetlands and back-swamps with aquatic and/or hygrophilous ferns, 2) xeric habitats dominated by Anemiaceae, and other drought-tolerant ferns, cheirolepids, gymnosperms including some Ginkgoales and ephedroids, and, probably, more local populations of Araucariaceae, 3) coastal/?lagoonal communities comprising *Classopollis*-producers, 4) frequently disturbed environments in which ferns and angiosperms occurred in varying magnitudes. The variability of depositional settings, based on the palaeobotanical and palynological proxies, is also corroborated by sedimentological data, that hitherto established the sedimentary environments as marine-influenced estuarine/deltaic [19,21,62,152].

#### 5.2.5. Charcoal

Relatively small fragments of macro-charcoal are abundant in six discrete levels in the type section of the Bahariya Formation at the Gebel El Dist succession, associated with non-charred wood fragments [8]. Charcoal originating from ferns was the most abundant in a single level and its anatomical details agree with the anatomy of *Paradoxopteris stromeri* which represents the rachis of the mattoniaceous fern *Weichselia reticulata*. Also, conifers and (putative) angiosperms were identified in the other levels analysed by [8].

The tree fern *W*. *reticulata* represents a taxon that is known to grow in habitats regularly influenced by fire, flooding, or other disturbances such as grazing by animals [187]. Moreover, *W*. *reticulata* also resembles a significant component of coastal vegetation from the Early to early Late Cretaceous (e.g., [31,34,60,123,124,128]).

Within the Gebel El Dist profile, charred ferns from the charcoal level 3 (Chl 3) were possibly growing in an analogous coastal (mangrove or mangrove-like) ecosystem, as it has been similarly presumed for abundant fern-charcoal deposits probably related to *Paradoxopteris* from the Barremian of Jordan [127]. A number of authors (e.g., [34,124]) construed *Weichselia*/*Paradoxopteris* as a representative component of mangrove-like vegetation, thriving during the Early-early Late Cretaceous. Nevertheless, this charcoal level at the Bahariya Oasis has to be viewed as allochthonous, and its origin from mangrove-like vegetation is still speculative. Bearing upon the previous discussion, the occurrence of microcharcoal, as black debris and opaque phytoclasts, has repeatedly been recognized from the studied subsurface material within the Bahariya Formation. This is also confirmed by previous palynological records from the same rock unit from different subsurface wells within the north Western Desert (e.g., [18,188]).

Lightning strikes within mangroves can lead to rather restricted patches of dead trees [127], even without setting the plants on fire [189,190]. This could lead to spatially limited accumulations of fuel. The latter could simply be ignited by lightning during substantial dry spells, even in a generally humid climate. Moreover, an additional factor that might have promoted the ignition and spread of fires, despite the seasonally dry conditions, is atmospheric oxygen concentrations which were higher than today, as evidenced by different quantitative findings (e.g., [191]). As a result, it is possible to envisage coastal ecosystems (i.e., mangrove and/or tidal marshes, etc.) dominated by *W*. *reticulata* that experienced repeated wildfires, during the deposition of the Cenomanian Bahariya Formation.

In summary, the data presented by [8] together with our palynological data indicate that the recorded wildfires possibly affected different vegetation types, perhaps extending into the wet-end member of coastal ecosystems, fern-dominated mangroves (or mangrove-like vegetation).

One can conclude from the ecosystem development point of view that such fire actions frequently affected the landscapes at the northern shore of Africa during the Cenomanian and that they were essential sources of disturbance in palaeoenvironments in which carnivorous dinosaurs, such as *Spinosaurus* and *Carcharodontosaurus* roamed, e.g., [192]. Though it is still unclear how wildfires impacted those faunas, nonetheless, it should be clear that more or less regular fires contributed to ecosystem structure and function during the Cenomanian, as they do in many modern ecosystems (e.g., [193]).

#### 5.2.6. Climatic inferences

The Cretaceous was a time of warmer temperatures than today up to high latitudes [111]. Oxygen isotope data indicate that a warm global climate settled throughout the late Albian to late Cenomanian [184,194,195]). Atmospheric CO_2_ levels measured 4–5 times higher than today, probably due to rigorous volcanism (e.g., [2,184,196]). Sea level attained 100–200 m higher than at present, hence widespread coastal areas were flooded by shallow seas ([197,198]). Such circumstances may have had an effective role in the spreading of biota, tolerating migration and movements of warmth-favouring taxa to higher latitudes, and the occurrence of typical Northern Gondwana pollen taxa (e.g., *Schrankipollis*, *Brenneripollis*, *Pennipollis*) in the austral tip of South America, which gives a piece of evidence for this postulation [111].

The most common method to reconstruct humidity vs. aridity utilizing palynomorphs is to use the abundant indicator species of pollen and spores [81–83]. Aridity indicator species include ephedroids and *Classopollis* as the former ephedroids are affiliated with modern *Ephedra* [199] and *Welwitschia* [200]. The recent *Ephedra* is also usually considered a xerophytic element including shrubs with reduced leaves in the steppe, semi-desert, and similar dry habitats [82]. Their high abundance (i.e., *Ephedripites*) in the Abu Ballas Formation suggests dry hinterland environments, a viewpoint supported by clay mineralogical content [201]. Comparable ecological similarities are proposed for Mesozoic ephedroids and *Classopollis* due to their plentiful occurrence in evaporitic or red sediment sequences (see examples in [82]).

*Classopollis* are the pollen grains of the family Cheirolepidiaceae which have been associated with dry and/or saline settings [153], similarly, [202] suggested the existence of mangrove and salt marsh vegetation with cheirolepid association in the Cenomanian of Charentes, France. Cheirolepidiaceae may indicate dry climates as their macrofossils have xerophytic adaptations like reduced, scale-like leaves, thick cuticles, and sunken stomata [129].

Indicators that suggest a humid climate comprise fern spores [81]. Though, few Cretaceous fern species (e.g., *Onychiopsis* and *Weichselia*), are recognized to have xerophytic characteristics, for instance, reduced pinnules and sporangia fixed in protective tissues [1,203]. To judge which spore types were humidity indicators during the Cenomanian, we analysed the palynological content of four subsurface borehole deposits. The high proportion of *Classopollis*, together with the presence of Elaterates, and ephedroids, may indicate more arid conditions. Nevertheless, considering the diversity and wide distribution of *Classopollis* and ephedroids, it is probable that both occupied a substantial range of different environments, including coastal habitats (e.g., [151,204]). Besides charcoal, which probably indicates seasonal dryer conditions or infrequent drier spells, the co-occurrence of xerophytic elements of ephedroids and *Classopollis*, suggests relatively dry conditions, and this could infer deposition in a warm, dominantly seasonally dry subtropical climate [205].

However, the concurrent presence of humidity-loving palynoflora (including mainly Cyatheaceae and Schizaeaceae) cannot be disregarded. Moreover, most Mesozoic ferns (here represented mainly by *Cicatricosisporites*, *Concavissimisporites*, *Cyathidites*, *Deltoidospora*, *Dictyophyllidites*…etc., in addition to water ferns) may have grown under moist, rather warm conditions either in marshes, along river banks or as understorey in forests [206]. Most of these taxa in addition to Cycadales (such as *Cycadopites*, and *Monosulcites*) indicate drier and ‘warmer’ lowland circumstances [80]. In addition, the occurrence of *Pediastrum* and *Botryococcus* can be used to infer freshwater palaeoenvironments, where the water depth is shallow lacustrine, paludal, or low gradient fluvial [85].

Higher percentages of fern spores, together with the high abundance and diversity of Schizaeaceae indicate more humid conditions [207]. However, although aridity rises, a high percentage of *Afropollis*, possibly related to humid tropical climates is recorded [151]. The values of *Afropollis jardinus* are conversely proportional to the values of dinoflagellates. Such a correlation of *A*. *jardinus* is previously well reported from Gabon, Brazil, and also Spain (see discussion in [207]).

Additionally, the humidity indicators versus aridity indicators relationship shows that humidity indicators were dominant in most of the studied samples reaching up to 30.5%, 21.9%, 28.3% average abundance throughout the chronostratigraphic sequence of the Bahariya Formation in the studied GPJ-1, TSW-21 and GPTSW-7 wells, respectively (Table 4). However, lesser values are recorded (12.5%, 6.0%, 12.5%) throughout the Bahariya Formation in the studied GPJ-1, TSW-21, and GPTSW-7 wells, respectively (Table 4). Results from the GPT-3 are inconclusive as the total palynomorph counts never exceeded 100 grains (Table 2).

The recorded palynofloras include *Elaterosporites*, *Elateroplicites*, *Elaterocolpites*, *Senegalosporites*, *Sofrepites*, *Steevesipollenites*, *Afropollis*, *Cretacaeisporites*, and *Stellatopollis*. These taxa show a remarkable similarity and represent part of the palynofloral composition of the ASA Province [106] or its updated equal Albian-Cenomanian Elaterates Province of [29], inferring arid to a semi-arid and warm climate.

From the distribution of xerophytic and hygrophilous (i.e., spores) sporomorphs, several dry and arid phases associated with minor humid pulses could be outlined during the Cenomanian of Egypt, which is previously reported for the Cenomanian and Albian-Cenomanian of NE Africa (e.g., [82]).

Moreover, the existence of charcoal as evidence of wildfires supports the interpretation of a seasonal climate or a climate with repeatedly occurring dry spells. Built upon previous palynological studies, some contributions assumed a tropical warm, and semi-arid climate throughout the deposition of the Bahariya Formation (e.g., [140]). Such climatic conditions, with a clear and long dry season, can foster the ignition and spread of wildfires (e.g., [193]), particularly when the atmospheric oxygen concentration is supposed to be higher than present [208].

## 6. Conclusions

A. The present paper originates from a palynofloral and megafloral investigation of the Cenomanian strata retrieved from four boreholes, two surface exposures, and a collection of material retrieved from the Western Desert, Egypt, stored at the Museum für Naturkunde in Berlin, Germany. This has resulted in the recovery of well-preserved sporomorph assemblages, which possibly reflect a dynamic history of the parent vegetation. Palynofloras unambiguously represent a mixed association of ferns, conifers, monosulcates, Gnetales, as well as a diverse group of angiosperms. The integration of megafloral data with the palynofloral evidence facilitates the delineation of five main vegetation types on the basis of taxa occurring in the Cenomanian (Late Cretaceous) strata. These can be summarized as follows:

1. Swamp communities are dominated by aquatics such as Nelumbonaceae and water ferns (Salviniaceae and Marsileaceae) as well as halophytic monocots and a substantial number of arboreal elements (morphotypes 12 and 13).
2. Riparian arboreal communities dominated by angiosperms (morphotypes 7 and 8) possibly with hygrophilous ferns in the understorey.
3. Open landscapes dominated by herbaceous plants (probably including ferns) and low scrubs.
4. Representatives of the Araucariaceae, Cycadales, Gnetales, and possibly Ginkgoales and some angiosperms may have produced a kind of forest growing on nutrient-poor soils formed on sand. The understoreys of these forests could have encompassed spore-producers (Anemiaceae) capable of tolerating dry/nutrient-poor sites.
5. Coastal/?lagoonal groups comprising plants adapted to periodic flooding by saline or brackish waters, such as “mangrove” *Weichselia*. Cheirolepids (i.e., *Classopollis*) and Ginkgoales probably also occurred in such environments as reported from Europe.

B. The heterogeneous compositions of the palynofloral assemblages may be attributed to extensive contributions from producers within the local vegetation during the early Cenomanian of Egypt. The presence of different depositional environments ranging from fluvial to lacustrine or lagoonal in a generally arid climate could also have played an important role, along with an occasional slight marine influence based on the low recovery of dinoflagellate cysts. The general aspect of the sedimentation is consistent with accumulation in a shallow shelf environment during the early stages of the Late Cretaceous transgression.

C. Previous evidence from charcoal recovered from the studied succession in Gebel El Dist indicates that different types of vegetation may have been affected by fires, and this could be a source of ecological disturbance. Mangrove-like coastal vegetation, dominated by ferns comparable to *Paradoxopteris/Weichselia reticulata*, may have experienced fires during the deposition of the Bahariya Formation during excessive dry spells.

D. Comparison of the detailed records of sporomorphs, plant macro-remains, and charcoal assemblages from the studied successions offers new insights into the palaeoclimatic and palaeogeographic settings during the Cenomanian and Albian-Cenomanian transition in the north Western Desert, an area of immense economic interest for petroleum and groundwater resources in Egypt. Moreover, the studied Bahariya Formation and its equivalent rock units represent a landscape where iconic dinosaurs and associated animals roamed. It is important therefore to improve the understanding of Egypt’s Cenomanian climate. Many previous contributions have interpreted the vegetation only in a general context as belonging to the Albian-Cenomanian Phytogeographic Province (sensu Herngreen et al., 1996) and its equivalents from the coastal basins of West Africa and South America, but without employing climatic proxies or data. Our reconstructions based on detailed new analyses and newly integrated data reveal a generally warm and humid climate, punctuated by repeated phases of considerably drier conditions of varying duration.

## Supporting information

S1 File List of the recorded palynomorph taxa (arranged alphabetically), GPJ-1, TSW-21, GPT-3, and GPTSW-7, north Western Desert, Egypt. (DOC)
