# Correction: Vegetation and climate change at the southern margin of the Neo-Tethys during the Cenomanian (Late Cretaceous): Evidence from Egypt

**DOI:** 10.1371/journal.pone.0307337

**Published:** 2024-07-12

**Authors:** The PLOS ONE Staff

The ORCID iD is missing for the first author. Please see the author ORCID iD here:

- Author Haytham El Atfy’s ORCID iD is: https://orcid.org/0000-0003-1618-7220 (https://orcid.org/0000-0003-1618-7220).

Additionally, the last 23 columns of Table 2 are incorrectly merged. The original version of Table 2 is provided here as S1 Table.

The publisher apologizes for the errors.

## Supporting information

S1 Table Relative abundances of palynomorphs recorded from the Bahariya Formation in the studied wells, north Western Desert, Egypt. (XLSX)
