# Supplementary material for: Vegetation and climate change at the southern margin of the Neo-Tethys during the Cenomanian (Late Cretaceous): Evidence from Egypt
Source: PLoS One. 2023 Jan 30;18(1):e0281008. doi: 10.1371/journal.pone.0281008 (PMC9886267; doi:10.1371/journal.pone.0281008)
Supplement: S1 File — (DOC) [file pone.0281008.s001.doc]

Haytham El Atfy, Clément Coiffard, Salah Y. El Beialy, Dieter Uhl: Vegetation and climate change at the southern margin of the Neo-Tethys during the Cenomanian (Late Cretaceous): Evidence from Egypt

**S1. List of the recorded palynomorph taxa (arranged alphabetically), GPJ-1, TSW-21, GPT-3, and GPTSW-7, north Western Desert, Egypt.**

**I. Algae**

*Botryococcus* cf. *B. braunii*

*Pediastrum* spp.

*Schizosporis reticulatus*

**II. Spores**

*Ariadnaesporites* spp.

*Cibotiumspora jurienensis* (Balme) Filatoff, 1975

*Cicatricosisporites minutaestriata* Bolkhovitina, 1961

*Cicatricosisporites* spp.

*Concavisporites* spp.

*Crybelosporites pannuceus* (Brenner) Srivastava, 1977

*Cyathidites australis* Couper, 1953

*Cyathidites minor* Couper, 1953

*Gabonisporis vigourouxii* Boltenhagen, 1967

*Leptolepidites* spp.

*Matonisporites sp.*

*Trilobosporites laevigatus* El Beialy, 1994

*Triplanosporites* spp.

Indeterminate trilete forms

**III. Gymnosperms**

*Araucariacites australis* Cookson ex. Couper, 1953

*Balmeiopsis limbatus* (Balme) Archangelsky, 1977

*Classopollis* *brasiliensis* Herngreen, 1975

*Classopollis* spp.

*Cycadopites carpentieri* (Delcourt and Sprumont) Singh, 1964

*Cycadopites nitidus* (Balme) De Jersey, 1964

*Cycadopites ovatus* Rouse, 1959

*Cycadopites* spp.

*Ephedripites jansonii* Muller, 1968

*Ephedripites* spp.

*Eucommiidites minor* Groot and Penny, 1960

*Eucommiidites troedssonii* Potonié, 1958

*Equisetosporites ambiguus* (Hedlund) Singh, 1983

*Exesipollenites* sp.

*Monosulcites minimus* Cookson, 1947

*Monosulcites* spp*.*

*Steevesipollenites binodosus* Stover, 1964

*Steevesipollenites* cf. *binodosus*

Elaterate Complex:

*Elaterocolpites castelainii* Jardiné & Magloire, 1965

*Elateroplicites africaensis* Herngreen, 1973

*Elaterosporites klaszii* (Jardiné & Magloire) Jardiné, 1967

*Senegalosporites petrobrasi* Herngreen, 1974

*Sofrepites legouxae* Jardiné, 1967

**IV. Angiosperms**

*Afropollis jardinus* (Brenner) Doyle et al., 1982

*Afropollis kahramanensis* Ibrahim & Schrank, 1995

*Albertipollenites* *rosalindiae* Srivastava, 1969

*Cretacaeiporites aegyptiaca* Ibrahim et al., 2015

*Cretacaeiporites densimurus* Schrank & Ibrahim, 1995

*Cretacaeiporites polygonalis* Herngreen, 1974

*Cretacaeiporites scabratus* Herngreen, 1974

*Dichastopollenites dunveganensis* Singh, 1983

*Dichastopollenites ghazalatensis* Ibrahim, 1996

*Foveomorphomonocolpites rashadi* Ibrahim, 1996

*Foveotricolpites giganteus* (Jardiné & Magloire) Jan du Chêne et al., 1978

*Foveotricolpites gigantoreticulatus* (Jardiné & Magloire) Schrank, 1987

*Integritetradites* *porosus* Schrank & Mahmoud, 2000

*Monocolpopollenites* spp*.*

*Nyssapollenites* sp.

*Pennipollis peroreticulatus* (Brenner) Friis, Pedersen & Crane, 2000

*Proteacidites* cf. *africaensis* (Jardiné & Magloire) Schrank & Ibrahim, 1995

*Retimonocolpites variplicatus* Schrank & Mahmoud, 1998

*Retimonocolpites* spp.

*Retitricolpites* sp.

*Retitricolporites pristinus* Singh, 1983

*Rousea* sp.

*Stellatopollis* sp.

*Syncolpites* spp.

*Tetracolpites* spp.

*Tricolpites* spp.

*Tricolporopollenites* spp*.*

**V. Dinoflagellate cysts**

**VI. Acritarchs**

**VII. Fungal palynomorphs**

**X. Miscellaneous**

Microforaminiferal test linings

Scolecodonts
